# Dual-Oriented Targeted Nanostructured SERS Label-Free Immunosensor for Detection, Quantification, and Analysis of Breast Cancer Biomarker Concentrations in Blood Serum

**DOI:** 10.3390/bios15070447

**Published:** 2025-07-11

**Authors:** Mohammad E. Khosroshahi, Christine Gaoiran, Vithurshan Umashanker, Hayagreev Veeru, Pranav Panday

**Affiliations:** 1 Nanobiophotonics & Biomedical Research Laboratory, M.I.S. Electronics Inc., Richmond Hill, ON L4B 1B4, Canada; christinegaoiran@gmail.com (C.G.); vithurshanumashanker@gmail.com (V.U.); hayagreev.veeru@uwaterloo.ca (H.V.); pranav.panday@uwaterloo.ca (P.P.); 2 Institute for Advanced Non-Destructive and Non-Invasive Diagnostic Technologies (IANDIT), University of Toronto, Toronto, ON M5S 3G8, Canada; 3 Department of Mechanical and Industrial Engineering, University of Toronto, Toronto, ON M5S 3G8, Canada; 4 Department of Electrical and Computer Engineering, University of Waterloo, Waterloo, ON N2L 3G1, Canada; 5 Department of Nanotechnology Engineering, University of Waterloo, Waterloo, ON N2L 3G1, Canada

**Keywords:** breast cancer biomarkers, concentration quantification, surface-enhanced Raman scattering, directional antibody, gold nanourchin

## Abstract

In clinical applications of surface-enhanced Raman spectroscopy (SERS) immunosensors, accurately determining analyte biomarker concentrations is essential. This study presents a non-invasive approach for quantifying various breast cancer biomarkers—including human epidermal growth factor receptor II (HER-II) (2+, 3+ (I), 3+ (II), 3+ (III), and positive IV) and CA 15-3—using a directional, plasmonically active, label-free SERS sensor. Each stage of sensor functionalization, conjugation, and biomarker interaction was verified by UV–Vis spectroscopy. Atomic force microscopy (AFM) characterized the morphology of gold nanourchin (GNU)-immobilized printed circuit board (PCB) substrates. An enhancement factor of ≈ 0.5 × 10^5^ was achieved using Rhodamine 6G as the probe molecule. Calibration curves were initially established using standard HER-II solutions at concentrations ranging from 1 to 100 ng/mL and CA 15-3 at concentrations from 10 to 100 U/mL. The SERS signal intensities in the 620–720 nm region were plotted against concentration, yielding linear sensitivity with R^2^ values of 0.942 and 0.800 for HER-II and CA15-3, respectively. The same procedure was applied to breast cancer serum (BCS) samples, allowing unknown biomarker concentrations to be determined based on the corresponding calibration curves. SERS data were processed using the *filtfilt* filter from *scipy.signal* for smoothing and then baseline-corrected with the Improved Asymmetric Least Squares (IASLS) algorithm from the *pybaselines.Whittaker* library. Principal Component Analysis (PCA) effectively distinguished the sample groups and revealed spectral differences before and after biomarker interactions. Key Raman peaks were attributed to functional groups including N–H (primary and secondary amines), C–H antisymmetric stretching, C–N (amines), C=O antisymmetric stretching, NH_3_^+^ (amines), carbohydrates, glycine, alanine, amides III, C=N stretches, and NH_2_ in primary amides.

## 1. Introduction

The development of sensitive diagnostic techniques for point-of-care applications is both essential and challenging in the context of early cancer detection. This need is increasingly urgent due to the growing emphasis on minimizing unnecessary biopsies of benign tissues, which can lead to physical discomfort, psychological stress, and increased healthcare costs. Ideal diagnostic tools should be non-invasive, cost-effective, easy to use, and capable of delivering rapid and reliable results. Cancer is a highly complex disease, driven by genomic abnormalities and molecular alterations. It is characterized by uncontrolled cell proliferation and chromosomal instability. Globally, cancer remains one of the most concerning public health challenges, contributing significantly to morbidity and mortality across populations [1,2]. As an example, breast cancer (BC) is the most prevalent neoplasm in women and the second leading cause of global mortality [3,4]. As of 2022, the most recent year for which global data are available, BC remains the most commonly diagnosed cancer worldwide and the leading cause of cancer-related deaths among women. According to the World Health Organization and World Cancer Research Fund International, approximately 2.3 million women were diagnosed with BC globally in 2022, making it the most prevalent cancer among women. In the same year, an estimated 670,000 women died from BC, accounting for 6.9% of all cancer-related deaths worldwide. If current trends continue, by 2050, the number of new breast cancer cases is expected to rise by 38%, reaching 3.2 million annually, and deaths are projected to increase by 68%, totaling 1.1 million per year. The BC subtypes are commonly grouped into four categories based on the immunohistochemical expression of hormone receptors: estrogen receptor-positive (ER+), progesterone receptor-positive (PR+), and human epidermal growth factor receptor-positive (HER-II+). HER-II genes make HER-II proteins, which are receptors on breast cells that help control cancer growth. HER-II overexpression is one of the earliest events in breast carcinogenesis and is considered to be a promising real-time biomarker for the presence or recurrence of tumors. In contrast, triple-negative breast cancer is characterized by the absence of expression of HER-II, estrogen, and progesterone receptors, making it more challenging to detect and treat using receptor-targeted therapies [5,6]. CA 15-3 is a carbohydrate-containing protein antigen from mucin (MUC), which is a large transmembrane glycoprotein. MUC1 (e.g., CA 15-3, MCA, CA 549, CA 27.29) is a protein family secreted by the luminal surface of glandular epithelia and has been used for early detection and prognosis in pre-diagnosis samples [7,8]. The MUC1 gene is overexpressed in malignant breast tumors, allowing the use of the gene product CA 15-3 as a tumor marker for BC [9], and the serum level of CA 15-3 corresponds to the tumor size. CA 15-3 is the most commonly used biomarker in breast cancer, with levels typically rising above 30 U/mL in affected patients [10]. Therefore, the ability to detect CA15-3 at low concentrations using SERS has significant implications for early breast cancer diagnosis and monitoring.

Early detection through regular screening and advancements in treatment have contributed to improved survival rates. Conventional screening techniques such as biopsy, mammography, magnetic resonance imaging (MRI), computed tomography (CT), positron emission tomography (PET), single-photon emission computed tomography (SPECT), and ultrasound can be invasive, costly, and in some cases destructive. Many of these methods involve exposure to ionizing radiation or the use of radioactive and contrast agents, such as gadolinium-based compounds and iodine-containing substances. Additionally, they often require specialized facilities and trained personnel for the safe handling of radiopharmaceuticals. Additional limitations and technical challenges include insufficient sensitivity, limited selectivity, and suboptimal spatial resolution. These shortcomings can increase the risk of both false-positive and false-negative results, potentially leading to delayed diagnosis or unnecessary interventions, each of which may have significant psychological impacts on the patient. A biomarker is a biochemical substance that acts as an indicator of physiological conditions, which can be in the following forms: (a) cellular, associated with the tumorous cells, or (b) humoral, secreted either by the tumor itself or during the tumor’s disintegration into the biofluid [7,11]. Blood-based biomarkers have significant importance in non-invasive, rapid, real-time, and accurate cancer screening, therefore providing information about early-stage tumor development, improving cancer patients’ survivability, and reducing the total national economic burden [7].

Optical biosensors offer a powerful alternative to traditional analytical methods, with significant potential for the direct, real-time, and label-free detection of various chemical and biological substances. Label-free biosensors detect target analytes by leveraging their intrinsic biophysical properties—such as electrical signals, refractive index, and charge distribution. The primary goal of biosensors is to generate an electronic signal whose intensity or frequency is proportional to the concentration of a specific analyte or group of analytes [12]. Raman spectroscopy is a powerful optical technique that provides a chemical fingerprint of the analyte based on Raman scattering, based on the inelastic scattering of photons, to measure the molecular transition energy resulting from the interaction between incident light and a molecule, leading to molecular vibration. This interaction results in a measurable energy shift corresponding to molecular transitions. The resulting frequency shift, expressed in wavenumbers (cm^−1^), is unique to each molecular species and reveals detailed information about the sample’s molecular composition and functional groups. The intensity of these Raman shifts is directly proportional to the number of scattering molecules interacting with the light. In cancer diagnostics, Raman shifts can be utilized to identify biochemical differences between healthy and tumor tissues. Cancerous tissues typically exhibit elevated concentrations of proteins, lipids, nucleic acids, and carbohydrates, offering a biochemical basis for potential diagnostic applications [13,14]. However due to very weak signals, they need to be improved via SERS, which is based on the coherent collective oscillation of conduction-band electrons in plasmonic nanoparticles (PNPs) when subjected to an external electric field. The PNPs exhibit strong optical absorption and scattering due to localized surface plasmon resonance (LSPR) (i.e., non-propagating surface plasmons) [14,15]. In contrast to LSPR, when the frequency of light waves matches that of surface plasmons, light waves can couple, excite, and propagate the surface plasmons at the metal–dielectric interface, called propagating surface plasmon resonance or surface plasmon polaritons (SPPs). There are two main mechanisms for SERS: The first is an electromagnetic (EM) field where the plasmon oscillation induces a dipole moment, which, in turn, couples and amplifies the local electric field at the frequency ω on the surface of a PNP due to LSPR, resulting in an intensified light absorption and scattering at the frequency of the surface plasmon resonance (SPR) [16]. The second mechanism is a chemical enhancement process via charge transfer between molecules and NPs, wherein the target analyte chemically bonds with the PNP surface. This bonding leads to the mixing of analyte–PNP energy levels, resulting in an amplified Raman signal similar to the resonance Raman effect. There has been extensive research utilizing other techniques for cancer detection, including electrochemical [17,18] and fluorescence spectroscopy [19]. More recently, field-effect transistor-based methods, especially graphene-based field-effect transistors (GFETs), have been used for the detection of colorectal cancer-derived exosomes, which are small extracellular vesicles (30–150 nm in diameter) secreted by cells [20,21]. Therefore, it appears that the integration of label-free SERS and exosome-based diagnostics will improve the detection precision because it carries a rich cargo of proteins, lipids, and nucleic acids that reflect the physiological or pathological state of their originating cells, including tumor-derived exosomes. Thus, when label-free SERS is applied to exosomes, it can provide rich spectral fingerprints of their molecular contents in a non-destructive, highly sensitive, and rapid manner [20].

Among different nanostructures, GNUs, as anisotropic 3D nanocrystals, offer unique optical properties compared to spherical gold NPs of the same core size. This is owed to the multi-branched shape and uneven surface with various lengths, resulting in redshifts within the SPR peak. A GNU has several plasmon resonances associated with several individual tips, contributing to a significant amplification of the EM field at the branch tips within smaller and highly localized volumes [22]. As a result, a strong enhancement of light–matter interaction at a spatial scale comparable to the size of the hot spots is expected [23,24]. Therefore, the hybridization of the core plasmons and individual spikes leads to multiple plasmon resonances, providing more efficient biosensing or therapeutic applications due to ample hot spots and a higher degree of functionalization with targeting species [25,26].

Despite the advances in plasmonic materials, some limitations prohibit their wide applications, such as restriction of the signal enhancement in the close vicinity of the substrate surface due to the exponential decay of the electromagnetic field. Therefore, analytes located within this region and at hot spots will experience the SERS field enhancement limited to those outside the Raman-active region. One approach to this is the functionalization of nanostructures, so that the target analyte may be attracted to the surface of the plasmonic nanostructure. In addition, plasmonic SERS substrates comprising metal nanostructures such as GNUs placed on top of metal films, i.e., plasmonic-active nanostructured thin films (PANTFs), result in an increased field enhancement from both the film and GNU, interparticle plasmon coupling (hot gaps), and the high localized field at the tips of the GNU, which contribute to the overall increase in the Raman signal [27]. Precise control of the distance between nanostructures is vital for the reproducibility of the measurements, which is normally achieved via a molecular linker. The PANTF strength is influenced by the Coulomb interaction between the surface charges (i.e., LSPR) on the nanostructure and the SPPs, which are in the propagating mode at the metal surface.

When comparing detection techniques, ELISA is traditionally considered to be the gold standard due to its high reliability, suitability for clinical diagnostics, and widespread use in high-throughput screening with FDA-approved kits. However, it is label-dependent, time-consuming, and not easily adaptable for real-time or portable applications. Electrochemical sensors are emerging as cost-effective, sensitive, and portable alternatives, being particularly well suited for point-of-care diagnostics, wearable biosensors, and rapid onsite testing. They often require careful surface functionalization and calibration to maintain their specificity and accuracy. Label-free SERS offers significant advantages for rapid, multiplexed detection and chemically specific biomarker profiling, especially in cases where labels may interfere or multiple analytes must be monitored simultaneously. Despite its potential, challenges such as signal reproducibility and biofouling in complex biological matrices like serum still need to be addressed.

The development of an efficient, label-free SERS probe for fast, sensitive, and selective detection of low-abundance biomarkers in cancer diagnostics is feasible by surface functionalization and bioconjugation of the NPs using a variety of suitable targeting agents, such as monoclonal antibodies (mAbs), peptides, aptamers, and cell surface ligands [28,29,30,31,32,33]. However, for maximum efficiency and long-term stability of mAbs or other ligands, immunosensors are normally fabricated on solid substrates. Therefore, the advantages of a PANTF system include (a) producing both propagating SPPs and LSPR, and (b) a higher sensitivity due to coupling of the SPPs of the thin film and those of the nanostructures [34,35]. This can be used as a transducer for different types of plasmonic-based sensing techniques: propagating SPPs, localized PR, and SERS-based sensing methods. Since label-free immunosensing relies on the analyte detection after directly binding to the antibody immobilized onto the SERS substrate, the PANTF SERS-based immunosensor is expected to offer an overall better performance than that of thin films and NP-immobilized substrates alone, as mentioned above.

Directional conjugation of mAbs on the surface of the substrate or nanostructure enhances the binding efficiency of target molecules, thereby improving the overall sensor detection performance. This can be attributed to the increased accessibility of antigen-binding sites (epitopes) when mAbs are properly oriented, as opposed to randomly immobilized mAbs, which may hinder analyte recognition. Directionality is achieved via the fragment crystallizable (Fc) regions of antibodies that can establish highly stable hydrazone bonds with adipic acid dihydrazide (ADH) molecules, enabling precise orientation of the Abs while keeping the fragment antigen-binding (Fab) sites free for binding. The N-ethyl-N′-(3-dimethylaminopropyl) carbodiimide hydrochloride (EDC)/ADH hydrazone bond chemistry method offers a more efficient method for biomarker detection. This selectivity leads to improved availability of the Fab region and enhances the overall effectiveness of biomarker detection.

Therefore, the ability to rapidly and accurately quantify the concentration of an unknown breast cancer (BC) sample would provide significant clinical value by supporting early detection and enabling timely decision-making by physicians. It is important to emphasize, however, that SERS-based immunoassays are intended to complement and not to replace the existing imaging and screening techniques. Final diagnostic confirmation and further analysis should refer to standard methods such as immunohistochemistry (IHC) and fluorescence in situ hybridization (FISH), as deemed appropriate by the attending physician. Within this context, advancing research in non-invasive SERS-based diagnostics is essential to address critical gaps and deliver timely, clinically relevant information that can support early decision-making and potentially improve patient survival rates. However, it is equally important to recognize that such technologies are not, by definition, authorized to independently determine a patient’s cancer stage. Diagnosis and staging involve a complex interplay of biological and clinical factors that fall strictly within established medical protocols and must be assessed by qualified healthcare professionals.

Building on our recent work involving the detection of BC biomarkers in serum using colloidal-based approaches [36,37], substrate configurations [33,34,35,36,37,38], and PANTF platforms [39], we propose the following objectives to extend our previous research: (a) perform SERS-based detection of several purified HER-II and CA15-3 breast cancer biomarker samples with known concentrations; (b) construct a calibration curve using data from (a), supported by an in-house-developed Python 3.12 software program; (c) conduct SERS detection of HER-II and CA15-3 biomarkers in various BCS samples with unknown concentrations; and (d) quantify the concentration of unknown CA15-3 HER-II biomarkers by referencing the established calibration curve.

## 2. Materials and Methods

### 2.1. Reagents

Citric acid-stabilized 20 mL GNUs with 90 nm diameter, 5.37 × 10^9^ NP/mL, a weight concentration of 3.97 × 10^−2^ mg/mL, and a molar concentration of 8.92 × 10^−12^, dispersed in 0.1 mM phosphate-buffered saline, were purchased from Cytodiagnostics, Canada. Pure ethanol reagent alcohol (277649), 1,6-hexanedithiol (HDT), adipic acid dihydrazide (ADH), EDC, [2-(N-morpholino)ethanesulfonic acid hydrate (MES hydrate, M8250-25G), sodium phosphate (NaH_2_PO_4_), 30 kDA centrifugal filters (UFC503096, Millipore), sodium chloride (NaCl, MW = 58.44 g/mol), 37% hydrochloric acid (HCl), sodium periodate (NaIO_4_), ethanolamine (NH_2_CH_2_CH_2_OH, 72068-100 mL, MW = 61.09 g/mol), bovine serum albumin (BSA, A2153-10G), micropipette tips (Eppendorf, 2231302001), microcentrifuge tubes (OF-17710-11), and a combo pH meter BLU2300E, Canada) were purchased from Sigma (Oakville, Canada); 5k HS-PEG-COOH (PEG: polyethylene glycol) was purchased from Biochempeg (HE003019-5K) (Massachusetts, USA); 1× PBS pH 7.4, ultrapure distilled water, and 70% ethanol in water (BP82031GAL), all of analytical grade, were purchased from Fisher Scientific (Ottawa, Canada). A microcentrifuge, orbital shaker (RK-51700-13), vortex shaker (RK-04729-07), and analytical balance (Sartorius) were purchased from Cole-Palmer. In addition, a scintillation vial, ultrasonic bath (Elmasonic), and fume hood (ISOLA, Mystaire, Canada) were also used during the experiment. HER-II and CA15-3 standard solutions (i.e., pure) were purchased from R&D Systems (1129-ER-050, Minnesota, USA) and Lee Biosolutions (Missouri, USA), respectively. Serum samples from breast cancer (BCS) patients with known HER-II concentrations and CA15-3 status were purchased from both BioIVT (New York, NY, USA) and Precision for Medicine (Massachusetts, USA), as listed in Table 1. All chemicals and reagents were of analytical grade. HER-II and CA15-3 monoclonal antibodies (mAbs) were purchased from SinoBiological (10004-MM03, HER-II/ErB_2_/CD340 Antibody, Mouse Mab, and 12123-MM05, MUC1/Mucin 1/CD227 Antibody, mouse Mab, respectively; Pennsylvania, USA). Table 2 summarizes the characteristics of CA15-3 and the clinical status of the corresponding BCS samples.

### 2.2. Functionalization of Substrates

Several PCB substrates were coated with a 50 nm thin gold layer using the immersion technique as a single-step bath process. Before the immobilization, functionalization, and conjugation of GNUs, the substrates were placed inside a plastic container with 800 μL of 70% ethanol for cleaning. The container was sealed and sonicated (Elma, Germany) at 60 kHz and 60% power for 30 min to completely clean the gold surface. An ethanol rinse was repeated twice, and then the substrates were left to air-dry and stored at room temperature for further use. To prepare 15 pM concentrated GNUs, a volume of 8.92 pM of 90 nm GNUs was added to three separate microcentrifuge tubes—1.12 mL (tube I), 1.12 mL (tube II), and 1.13 mL (tube III)—inside the biosafety cabinet and microcentrifuged at 3200 rpm for 30 min. A fourth tube with 1.13 mL of water was also prepared for balance. The tubes were placed in a microcentrifuge set at 3200 rpm for 30 min. After centrifugation, the supernatant of the tubes was removed, leaving 25 μL of pellet from each tube. Then, 0.56 mL of ultrapure water was added to tubes I and II, and 0.565 mL was added to tube III. The entire volumes of resuspended GNUs were transferred to a 20 mL scintillation vial, giving a total of 1.69 mL of resuspended GNUs. A volume of 0.35 mL of ultrapure water was added to the vial for a final volume of 2.035 mL of 15 pM concentrated GNUs.

The concentrated GNUs were immobilized on a gold-coated PCB via an HDT (i.e., GNU-HDT) surface via gold–thiol bonds. First, 1 µL of HDT and 1999 µL of pure ethanol reagent alcohol were mixed in a 20 mL glass scintillation vial and capped immediately. The vial was vortexed at 1800 rpm, creating a 2 mL solution of 3 mM ethanolic HDT. The GNU-HDT substrate was then immersed in the prepared mixture and sonicated at 60 kHz and 60% power for 2 min to functionalize the gold surface with HDT molecules. Following the incubation, the substrate was sequentially rinsed with 70% ethanol and ultrapure water inside the biosafety cabinet. The functionalized substrate was incubated in 800 μL of prepared 15 pM concentrated GNU solution for 10 min at 150 rpm, permitting the binding of the GNUs to the free thiol end of the HDT. The substrate was rinsed with ultrapure water and air-dried in the biosafety cabinet.

The GNU-HDT was further functionalized with thiol- and -COOH-terminated PEG linkers (i.e., GNU-HDT-PEG). Then, 50 mg of 5k HS-PEG-COOH was weighed and added to a 20 mL scintillation vial containing 2 mL of ultrapure water. The vial was vortexed at 1800 rpm for 30 s to dissolve the PEG powder. The GNU-HDT-PEG was then placed in a container containing 800 μL of PEG solution. The container was capped and left to mix overnight on the orbital shaker set to 150 rpm at room temperature. The following morning, the substrate was rinsed with ultrapure water to remove any unbound PEG, air-dried, and then characterized with FTIR and Raman spectroscopy as described above. For the functionalization of PEG with ADH (i.e., GNU-HDT-PEG-ADH), inside the biosafety cabinet, 58.2 mg of ADH and 29.1 mg of EDC were weighed and added to a scintillation vial containing 2 mL of ultrapure water. The powders were dissolved by vortexing at 1800 rpm, yielding a 167 mM ADH and 76 nM EDC solution. Then, 800 μL of the prepared solution was added to a container containing the GNU-HDT-PEG-ADH. The container was capped and allowed to mix on the orbital shaker at 150 rpm for 3 h. Once the time was complete, the PCB was rinsed with ultrapure water to remove unbound ADH/EDC.

### 2.3. HER-II mAb Buffer Exchange

The next step involved performing a buffer exchange of the HER-II mAb from PBS to sodium phosphate buffer. Working within a biosafety cabinet, 14.19 mg of sodium phosphate monobasic (NaH_2_PO_4_) was weighed and dissolved in 1 mL of ultrapure water to prepare a 100 mM buffer solution. A 25 μL aliquot of 1 mg/mL HER-II mAb, originally supplied in PBS, was thawed and transferred into a 30 kDa molecular weight cutoff centrifugal filter. For balance, a second centrifugal filter was prepared containing 25 μL of ultrapure water. The filters were centrifuged at 14,000 rpm for 10 min. After centrifugation, the filtrate containing the PBS was discarded, while the mAb remained in the filter unit. To recover the mAb in the new buffer, 25 μL of the prepared 100 mM NaH_2_PO_4_ buffer was added to a clean microcentrifuge tube. The filter unit containing the concentrated mAb was inverted and placed into the recovery tube, followed by a reverse spin at 3000 rpm for 2 min. After centrifugation, the filter unit was discarded, and the microcentrifuge tube now contained 25 μL of HER-II mAb at 1 mg/mL in sodium phosphate buffer.

### 2.4. HER-II mAb Preparation

This stage involved several key steps: buffer preparation, purification, conjugation, stabilization, and blocking. A 10 mM sodium phosphate (NaH_2_PO_4_), 0.15 M sodium chloride (NaCl) buffer at pH 7.5 was prepared by dissolving 14.02 mg of NaH_2_PO_4_ and 87.68 mg of NaCl in 6 mL of ultrapure water in a 20 mL scintillation vial. The pH was adjusted to 7.5 using hydrochloric acid while monitoring with a calibrated pH meter. Once the desired pH was achieved, the solution was transferred to a 10 mL graduated cylinder and diluted to volume with ultrapure water. The final buffer was stored at 4 °C until use. Following buffer preparation, the HER-II mAb was activated using 0.1 M sodium periodate (NaIO_4_). Specifically, 2.14 mg of NaIO_4_ was dissolved in 100 μL of ultrapure water in a clean microcentrifuge tube, mixed thoroughly, and wrapped in aluminum foil to protect it from light. Then, 5 μL of this freshly prepared NaIO_4_ solution was added to 25 μL of HER-II mAb. The mixture was vortexed at 2200 rpm for 30 s and incubated on an orbital shaker at 275 rpm for 45 min. To quench the oxidation reaction, 200 μL of 1× PBS was immediately added. Finally, the original antibody buffer (~230 μL) was exchanged with 50 μL of the prepared coupling buffer using centrifugal filter units, following previously described protocols.

A total of 50 μL of HER-II mAb solution and 100 μL of the coupling buffer were added to a container containing the functionalized GNU-HDT-PEG-ADH substrate. The container was sealed and placed on an orbital shaker at 150 rpm to incubate overnight at room temperature. The following morning, under sterile conditions in a biosafety cabinet, 2 μL of 1 M ethanolamine was added to the substrate to stabilize the HDT linkage and prevent reversal of the bond between the ADH and the antibody. The reaction was allowed to proceed for 1 h at room temperature on the orbital shaker (150 rpm). To block any remaining reactive sites and reduce non-specific binding, a 10% (*w*/*v*) BSA solution was prepared by dissolving 6.0 mg of BSA in 60 μL of coupling buffer. Then, 15 μL of this blocking solution was applied to the surface of the PCB substrate and incubated for 10 min at room temperature on the orbital shaker (150 rpm). After incubation, the surface was washed with 1× PBS to remove excess BSA. At this stage, antibody conjugation to the nanostructured substrate was complete.

### 2.5. SERS Biosensor–Breast Cancer Serum Interaction

Frozen BCS samples were thawed overnight at 4 °C. For each test, 4 μL of thawed serum was mixed with 56 μL of 0.01 M PBS (pH 6) and 100 μL of MES buffer (pH 6) in a microcentrifuge tube, resulting in a total volume of 160 μL. The mixture was vortexed at 2200 rpm and then transferred onto the surface of the functionalized PCB substrate, ensuring complete coverage and immersion. The container was sealed and incubated on an orbital shaker at 150 rpm for 1 h at room temperature to facilitate analyte binding. After incubation, the substrate was rinsed thoroughly with MES buffer and air-dried before characterization by Raman and FTIR spectroscopy.

In addition to this sample, four additional biosensors were prepared, each exposed to a different BCS sample, as listed in Table 1. For calibration, a 100 µg/mL HER-II stock solution was prepared by reconstituting 50 μg of HER-II protein in 0.5 mL of 1× PBS (pH 7.4), following the manufacturer’s instructions. This stock was serially diluted using healthy human serum as the diluent to create ten standard solutions with concentrations ranging from 10 to 100 ng/mL. Ten PCB substrates were prepared—one for each concentration—using the same functionalization and sample incubation procedure described above. Each calibration point represents a unique, independently prepared substrate.

### 2.6. CA 15-3 Antibody Buffer Exchange, Activation, and Purification

A 50 µL aliquot of BCS antibody, initially supplied at 0.5 mg/mL in PBS, was first concentrated to 1 mg/mL. The PBS buffer was then exchanged for 25 µL of 100 mM sodium phosphate buffer using spin columns. Following buffer exchange, the antibody was activated by incubating with 5 µL of freshly prepared sodium periodate solution for 45 min at room temperature. To quench the reaction, 200 µL of 1× PBS was added. The activated antibody was subsequently purified, and its buffer was exchanged once again—this time into 50 µL of coupling buffer—using new spin columns. The coupling buffer consisted of 10 mM sodium phosphate and 0.15 M sodium chloride in ultrapure water, adjusted to pH 7.5.

### 2.7. CA 15-3 Antibody Conjugation

Following the functionalization of the PCB substrate with ADH and EDC, the substrate was placed in an air-tight container. To prepare the antibody solution, 100 µL of coupling buffer was added to 50 µL of the activated BCS antibody, resulting in a total volume of 150 µL. This solution was then evenly applied to the surface of the PCB substrate, ensuring complete coverage. The container was sealed and incubated overnight at room temperature to allow for covalent antibody immobilization. The next morning, 2 µL of 1 M ethanolamine was added directly to the substrate and incubated for 1 h at room temperature to stabilize the hydrazone linkage and prevent bond reversal. To minimize non-specific binding, the surface was subsequently blocked with 15 µL of 10% (*w*/*v*) BSA. Finally, the substrate was rinsed thoroughly with ultrapure water and prepared for further analysis.

### 2.8. Interaction of SERS Biosensor with CA 15-3 Standard Solution

**Figure 1:**
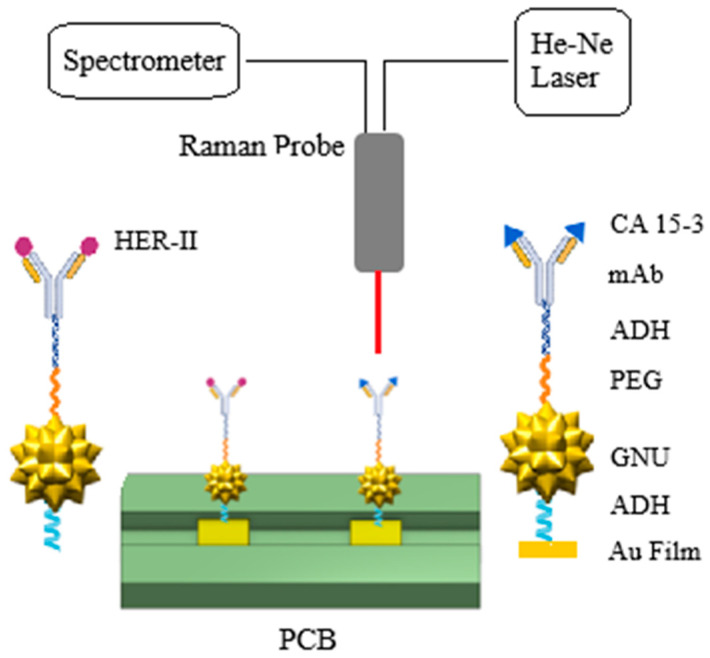
Schematic illustration of PCB-based double-active label-free immunosensor for detecting BCS HER-II and CA 15-3 biomarkers.

The BCS standard solution was originally supplied in aqueous form at a concentration of 16.5 kU/mL. This stock was serially diluted to prepare ten standard solutions with concentrations ranging from 100 to 10 U/mL. For each interaction experiment, 4 µL of the diluted BCS solution was mixed with 56 µL of PBS and 100 µL of MES buffer (pH 6), yielding a total volume of 160 µL. This solution was then applied to the surface of the PCB substrate, ensuring complete coverage and creating an immersed configuration for effective analyte binding. The substrate was incubated at room temperature for 1 h to allow for optimal interaction between the analyte and the immobilized antibody. The setup used for these experiments is illustrated in Figure 1.

### 2.9. Characterization Methods

UV–Vis characterization of GNU functionalization was performed using a Jenway 7205 spectrophotometer, operating over a spectral range of 198–800 nm. For SERS measurements, data were collected using OceanView 2.0 software with the following parameters: 5 s integration time, three scan averages, and a boxcar width of 5. Non-linearity correction was applied, and the “Clean Peaks” option was enabled. Raman excitation was provided by a benchtop 637 nm diode laser (S1FC637, Thorlabs, Newton, NJ, USA) set to an output power of 5 mW. The laser was coupled with a 638 nm Raman probe (RIP-RPB-638-FC-APC-SMA, Ocean Insight, (Gamble Technologies Limited), Mississauga, ON, Canada) with a 9.5 mm diameter. The probe contains a fiber-optic bundle that simultaneously delivers excitation light to the sample and collects Raman-scattered light. The system has a spectral detection range of 300–3900 cm^−1^ and a fixed focal distance of 7.5 mm. To reduce the background signal and detector overload, a dichroic edge filter was used to block Rayleigh-scattered light (at the laser wavelength) before it reached the spectrometer (HDX-Vis-NIR, Ocean Insight, USA). The dichroic filter covered a Raman shift range of 150–3400. An image was captured with the laser shutter closed against a dark background for reference.

## 3. Quantification

### 3.1. Calibration

To identify and quantify the BCS data points on the calibration curve, two approaches were evaluated: (a) selecting a single wavenumber corresponding to the strongest or most consistent overlapped SERS peak, and (b) identifying the most frequently occurring peaks within a defined spectral range. The first method presented challenges, including peak shifting and spectral non-uniformity across different samples. As a result, using a fixed wavenumber often failed to capture the true peak for all spectra, leading to potential misrepresentation of the data. The second method proved more robust, yielding a more accurate and consistent intensity–concentration relationship. By focusing on the most common peaks within a specified range, this approach effectively compensated for slight spectral shifts and improved the overall quantification accuracy. To implement this, a peak-finding algorithm with optimized parameters was used to isolate only the most prominent Raman peaks. The extracted peak intensities were then used to determine the analyte concentration by referencing the corresponding calibration curve.

### 3.2. Implementation: SpectraView (SV)

To eliminate unwanted signals and enhance the SERS spectral features, the data must undergo preprocessing to ensure reproducible qualitative and quantitative results before analysis. The essential preprocessing steps typically include (a) filtering, (b) baseline correction to remove spectral contributions from phenomena such as fluorescence, and (c) normalization to account for fluctuations, such as those from laser intensity. The experimental data were processed using SV, an in-house-developed Python-based software platform designed for analytical chemistry applications. SV allows users to visualize Raman spectroscopy data, such as those from HER-II biomarker analysis, and calculates the corresponding sample concentrations based on the SERS spectra. The software provides two main functions: (1) displaying the SERS spectra from the sample, and (2) calculating the concentration of the sample based on its spectral features. For the analysis, the software identifies the peak of interest in the range of 650–670 cm^−1^. It calculates the intensity of this peak, which varies depending on the specific biomarker, and then determines the corresponding concentration using the pre-established calibration curve. The spectral data are processed through several steps: they are frequency-filtered using the *filtfilt* function from *scipy.signal*, baseline-corrected with the *isals* function from the pybaselines.Whittaker library, and normalized using the minmax_scale function from *sklearn.preprocessing.* The intensity data are then used to determine the sample’s concentration. To extract the concentration, SV employs a peak-finding algorithm that locates the peak intensities in the region of interest. These intensities are input into a linear regression model to calculate the sample concentration, referencing the calibration curve of the selected pure biomarker. The current calibration curve predicts concentrations with reasonable accuracy, but it does not reliably predict values outside this range (below 10 ng/mL or above 100 ng/mL or U/mL). Importantly, SV analyzes the spectral range rather than relying on a single wavenumber, so as to avoid inaccuracies caused by small shifts in peak positions. The software uses data captured from the spectrometer and is integrated with proprietary software provided by Ocean Optics for data acquisition.

## 4. Results and Discussion

### 4.1. UV–Vis Spectroscopy

Before substrate fabrication, UV–Vis spectroscopy was employed to confirm the presence of specific molecules and functional groups throughout the functionalization and bioconjugation process. Proteins have characteristic UV–Vis absorbance peaks, often in the UV region, due to the aromatic amino acids that they contain.

**Figure 2:**
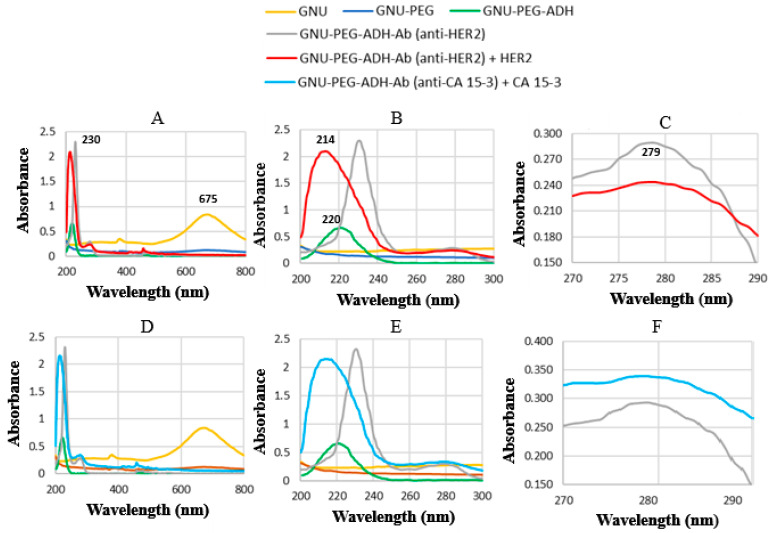
(**A**) UV–Vis spectra showing the stepwise functionalization and bioconjugation of gold GNUs exhibiting an LSPR peak at 675 nm, PEG-functionalized GNUs, ADH-functionalized GNU-PEG with a peak at 220 nm, and mAb-conjugated GNU-PEG-ADH displaying a peak at 230 nm followed by a characteristic peak at 214 nm after the interaction. (**B**) Magnified view of (**A**) between 200 and 300 nm. (**C**) Magnification of (**B**) between 270 and 290 nm, where the mAb shows higher absorbance than HER-II, and the corresponding results for CA 15-3 from (**D**–**F**). Note the higher absorbance of CA 15-3 than the mAb.

Figure 2A displays the UV–Vis spectra collected at various stages. The LSPR peak of the gold GNUs is visible, exhibiting a characteristic absorbance maximum at approximately 675 nm. Following surface functionalization with polyethylene glycol (PEG), the overall LSPR intensity decreased significantly, indicating successful surface modification. Normally, changes in the intensity or peak position of the UV–Vis absorbance spectra of gold nanoparticles and proteins can provide valuable information about their interactions, including protein binding, conformational changes, and aggregation. The intensity further decreased upon the covalent attachment of ADH, which also introduced a distinct absorption peak at 220 nm. This peak corresponds to peptide (amide) bonds, which typically absorb in the 190–220 nm range. Subsequent conjugation of mAbs to ADH resulted in two broad absorption peaks: one at ~230 nm, associated with a π → π* transition in the carbonyl group, and one occurring within the 190–230 nm range, reflecting strong absorbance from overlapping signals due to peptide bonds and aromatic residues such as phenylalanine (Phe).

Overlapping transitions often appear with antibody conjugation. The second peak observed at 279–280 nm primarily corresponds to aromatic amino acids such as tyrosine (Tyr) and tryptophan (Trp), used to quantify HER-II. The maximum absorbance was that of Trp, a key aromatic amino acid. The overall absorbance at 280 nm used in protein quantification depends primarily on the number of Trp and Tyr residues. The π → π* transition refers to the excitation of an electron from a bonding π orbital to an antibonding π* orbital, a process characteristic of double bonds (e.g., C=C, C=O), where π bonds arise from the lateral overlap of p orbitals. It is noteworthy that the absorbance of Trp can shift depending on various factors, including solvent polarity, hydrogen bonding, quenching effects from nearby residues (e.g., histidine, cysteine), and the overall protein conformation (e.g., folded vs. unfolded states) [40]. These transitions are crucial to understanding protein structure, dynamics, and detection using optical methods. Label-free detection methods like SERS and FTIR can sometimes detect signals influenced by these electronic transitions indirectly.

Following the interaction between the mAb and the HER-II biomarker, a distinct absorption peak appeared at 214 nm. HER-II is a receptor Tyr kinase, meaning Tyr residues are critical to its function, are present in multiple locations, and are especially important in the intracellular domain. HER-II also contains Trp residues, albeit in smaller numbers [41]. Upon binding to mAbs, or when immobilized on a substrate, peak shifts can occur. A peak around 214–220 nm is due to amide bonds (π → π*), indicating strong absorbance from a peptide backbone that is often used to track conformational changes or secondary structural features. These are not unique to HER-II but can help confirm molecular interactions in UV–Vis experiments, providing further evidence of successful biomolecular recognition. Figure 2B shows a magnified view of the spectra in the 200–300 nm range, where the peaks are resolved. The spectral overlap between the mAb and biomarker is further illustrated in Figure 2C, which shows a reduction in mAb absorbance following interaction, i.e., an effect attributed to biomarker adsorption. Similar trends can be observed for CA15-3 in Figure 2D–F. However, in Figure 2F, the absorbance of Trp is slightly higher than that of the mAb alone, suggesting lower biomarker adsorption during the interaction in this case. MUC1 is low in aromatic residues, but some absorbance near 280 nm may still occur if small amounts of Trp or Tyr are present in the MUC1 cytoplasmic or extracellular regions. The glycosylation of CA15-3 does not directly contribute to UV absorbance, but it can alter the environment around absorbing groups, potentially shifting or broadening the peaks slightly [42].

### 4.2. AFM of GNU-Immobilized Substrate

**Figure 3:**
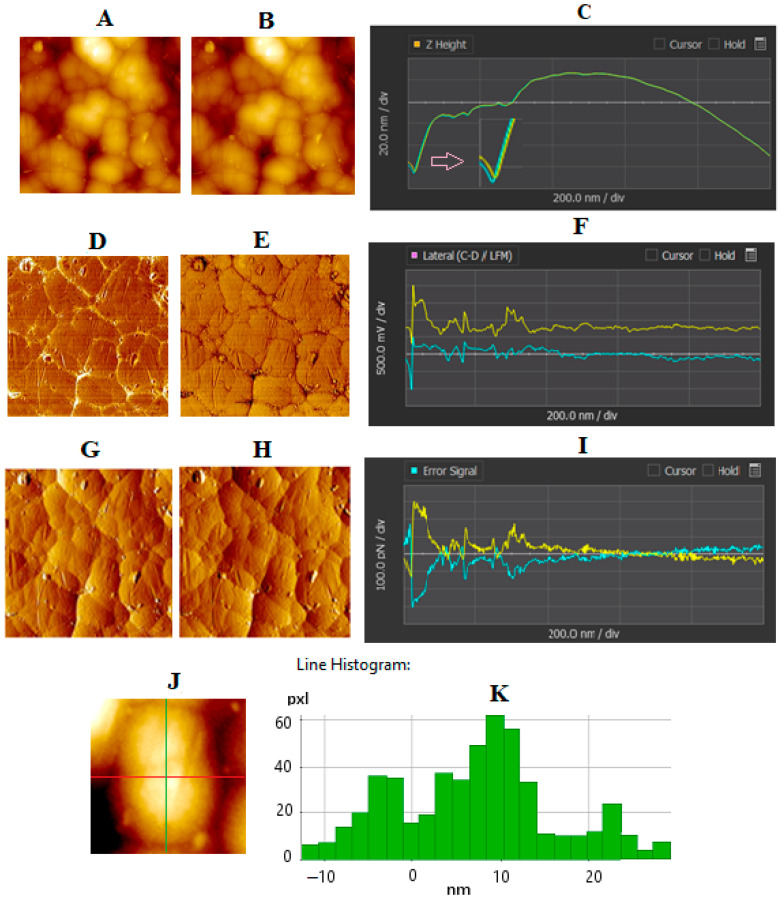
(**A**) AFM images and (**B**) z-height images of GNU-immobilized substrate in forward and backward scans; (**C**) plot of vertical displacement versus horizontal distance of the substrate surface; (**D**,**E**) the forward and backward lateral AFM images, respectively with the corresponding signal display in (**F**); (**G**,**H**) the forward and backward zero-error signals, respectively, with the corresponding signal display seen in (**I**); (**J**) the forward z-height of the substrate; and line profile showing a specific cross-section on the part with the corresponding histogram seen in (**K**).

The AFM images of the substrates were obtained in non-contact mode over a fixed area of 5 × 5 μm^2^ at a constant scan rate of 0.3 Hz, regardless of the imaging resolution. Figure 3A, B show the topographic (z-height) forward and backward scans, respectively, which display near-identical features, confirming the reproducibility of the measurements. These images represent vertical displacement (height) as a function of lateral position and were obtained by scanning a sharp cantilever-mounted tip across the sample surface in a line-by-line fashion. The tip records variations in attractive forces as it interacts with the sample, enabling high-resolution mapping of the surface morphology. The image contrast primarily arises from differences in surface height, where regions corresponding to GNUs appear brighter due to their elevated topography relative to the underlying substrate.

Figure 3C provides an example line profile showing the surface topography of the GNUs, where the height difference between the peak and the lowest point reaches approximately 80 nm (about 10 nm less than the average height reported for the supplied nanostructures) across a horizontal distance of 100 nm. Figure 3D,E present the corresponding forward and backward lateral AFM scans, with their respective detector signal traces shown in Figure 3F. During the forward scan, the laser beam shifts toward the positive side of the position-sensitive photodetector, resulting in a bright image contrast. Conversely, in the backward scan, the shift occurs in the opposite direction, producing a darker contrast [43]. Figure 3G,H illustrate the forward and backward zero-error signals, with their respective signal traces shown in Figure 3I. The error signal, output from the differential amplifier, reflects how accurately the probe tracks the surface contours. Ideally, when the probe perfectly follows the sample surface, the error signal approaches zero. Lastly, Figure 3J shows an example line profile that defines a specific cross-section of the surface, while the corresponding height distribution histogram is provided in Figure 3K. This quantitative analysis offers insight into the surface roughness and structural uniformity of the functionalized substrate.

The asymmetry of the roughness distribution can be tested by using the sample skewness, $R_{sk}$, which is a measure of symmetry over the surface profile, i.e., the lack of symmetry around the data point distribution curve. Other relevant parameters are $R_{q}$ (the root-mean-square average of height deviation taken from the mean image data plane) and $R_{a}$ (the arithmetic average of the absolute values of the surface height deviations measured from the mean plane). Based on the experimental data and the software, the corresponding values for $R_{q}$, $R_{a}$, and $R_{sk}$ are given in Table 3.

### 4.3. Enhancement Factor (EF)

**Figure 4:**
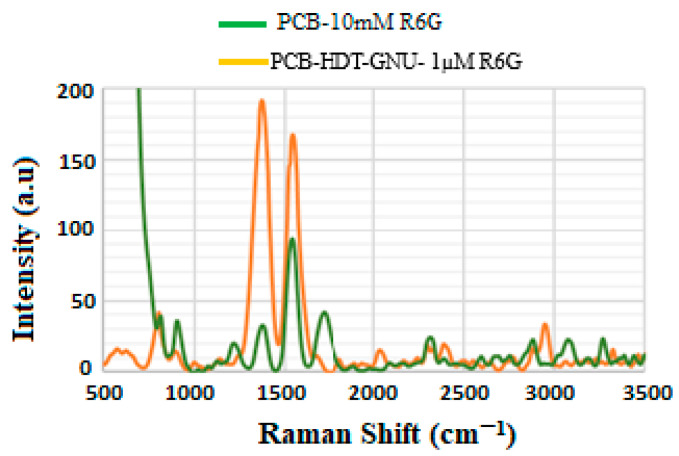
Measurement of EF for the nanobiosensor using R6G dye at 2 mW and 10 min incubation time.

To calculate the EF of the sensor, 10 μL of Rhodamine 6G (R6G) solution at concentrations of 10 mM and 1 μM was deposited onto both the PCB-coated thin gold film and the PANTF substrate. For the PANTF configuration, the applied volume of R6G provided coverage over a larger surface area due to the increased surface roughness and high density of the GNUs, which are known to enhance local electromagnetic fields. The EF was determined by comparing the intensity of characteristic Raman lines enhanced by LSPR using Equation (1), under a fixed laser power setting [44].

(1) $$EF=\frac{I_{SERS}}{I_{Raman}}\times \frac{C_{Raman}}{C_{SERS}}.$$

where $I_{Raman}$ is the intensity of the bare substrate with R6G at a concentration of $C_{Raman}$ = 10 mM, while $I_{SERS}$ is the intensity of GNU-immobilized substrate with R6G at a concentration of $C_{SERS}$ = 1 μM. Figure 4 presents the SERS results for the nanobiosensor at 2 mW and 10 min GNU incubation time.

According to Figure 4, $\frac{I_{SERS}}{I_{Raman}}=\frac{\langle \approx 190\rangle }{\langle \approx 40\rangle }$ and $\frac{C_{Raman}}{C_{SERS}}$ = 10^4^. Therefore, EF ≈ 0.5 × 10^5^.

The most prominent peaks are observed between approximately 1350 and 1550 cm^−1^, corresponding to C-C stretching in the xanthene ring, C-N stretching in NHC_2_H_5_, and C-C stretching in the phenyl ring [45,46].

### 4.4. SERS of Conjugation and Interaction of Standard HER-II Solution

The Raman spectroscopy is divided into three wavenumber regions: (a) a fingerprint region covering between 600 and 1800 cm^−1^, corresponding to abundant molecular vibration information such as characteristic peaks of proteins, lipids, carbohydrates, and nucleic acids; (b) a silent region, typically between 1800 and 2700 cm^−1^, featuring low Raman scattering intensity, mainly comprising molecular internal and lattice vibrations; and (c) a high-wavenumber region, ranging between 2700 and 3000 cm^−1^, encompassing the stretching vibration of C-H, O-H, N-H, and other chemical bonds, such as CH_3_ and CH_2_ stretching found in proteins, carbohydrates, lipids, and water [47,48]. Amino acids are divided into essential amino acids—including histidine (His), isoleucine, leucine, lysine, methionine, phenylalanine, threonine, tryptophan, and valine—and nonessential amino acids, including alanine, asparagine, cysteine, glutamine, glycine, proline, serine, and Tyr.

**Figure 5:**
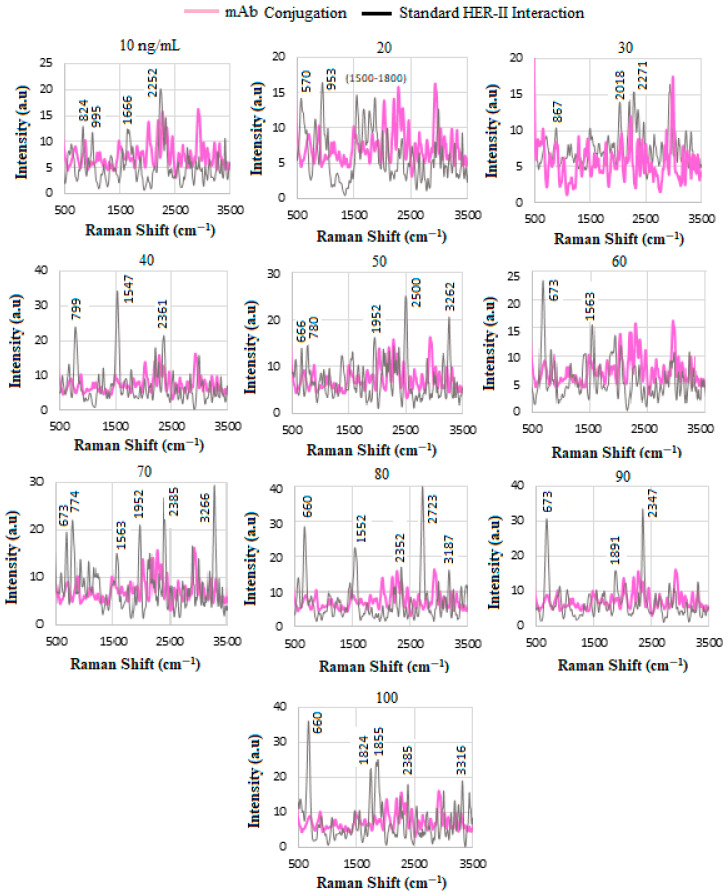
SERS signals of standard HER-II solution for before (mAb-conjugated) and after interaction at various concentrations.

Figure 5 indicates the comparison of the SERS spectra for the standard HER-II solution at various concentrations for conjugation and interaction. A characteristic Raman band in the amino acid family around the 500 cm^−1^ region is due to a disulfide bond (SS-bond), i.e., a disulfide bridge, which is a strong covalent bond between two sulfhydryl (–SH) groups. The exact location of the SS band is determined by the conformation of the residues contributing to the disulfide bridge and, thus, the protein’s three-dimensional structure [43]. Despite some guidelines in assigning bands to structural features, spectral shifts due to the sensitivity of bands to fine structural changes are possible, particularly in mixtures. The range between 500 and 700 cm^−1^ mainly pertains to proteins such as Tyr amino acids at 570 and 660 cm^−1^ [49,50]. The bands near 800, 824, and 867 cm^−1^ are thought to correspond to Tyr-containing proteins [49,51]. It was also suggested that the peaks at 820, 821, 870, and 871 cm^−1^ may correspond to isoleucine, Phe, threonine, and aspartic amino acids in solution [52]. Some carbohydrates can be observed between 700 and 1000 cm^−1^, such as D-(+) sucrose at 724 cm^−1^, D-(−) ribose at 824 cm^−1^, and D-(+)-raffinose pentahydrate at about 953 cm^−1^. Not much spectral information is observed between about 1000 and 1500 cm^−1^. Other dominant Raman lines between ≈1540 and ≈1570 cm^−1^, including 1547 and 1552, and 1563 cm^−1^, likely denote N-H primary, Trp, and secondary amines and amides, respectively, while those between 1645 and 1680 cm^−1^ correspond to α-helical domains in proteins contributing to amide I and amide III modes and β-sheet structures [51]. The lines between about 1800 and 2700 cm^−1^ are in the silent region, where mainly the structural value of the Raman thiol SH stretching bands such as 2347, 2500, and 2723 cm^−1^ is observed. The lines are influenced by the band frequency’s sensitivity to hydrogen bonding of the SH donor and S acceptor groups, along with the band intensity, which is a measure of the concentration of thiol groups in the protein [53]. At larger wavenumbers, the lines between 2850 and 3000 cm^−1^ are associated with highly overlapped bands that result from C-H stretching vibrations; those at 3000–3100 cm^−1^ to aromatic C-H stretching; 3100 cm^−1^ to a weak and broad band assigned to NH_3_^+^; 3110 and 3160 cm^−1^ to two sharp and intense bands of the imidazole heterocyclic ring structure of His; 3187 cm^−1^ to the presence of CH_3_, CH_2_, and =CH stretching in proteins; 3266 cm^−1^ to NH_3_^+^ I amine [30] 3316 cm^−1^ to NH_2_ in aromatic amines, primary amines, and amides; and 3330–3400 cm^−1^ to NH_2_ stretching in primary amines [32]. Some others have suggested that the lines between 2800 and 3100 cm^−1^ are related to CH_3_ and CH_2_ stretching in carbohydrates, lipids, and proteins [47].

The intensity of the SERS signals increases after interaction, due to the presence of additional proteins and carbohydrates in the biomarker, and becomes more pronounced as the concentration increases. However, the variation in the profile can be because of the presence of other biochemical compounds that characterize each biomarker sample, as well as the possible detachment of the biomarker from the mAb. It is known that the stronger and more intense signals originate from the hot spots with high field gradients, where the electromagnetic field is highly focused and rapidly propagates on the surface of PNPs at a higher rate due to the higher thermal conductivity of gold NPs than that in the surrounding medium (such as water) [54]. The micro-heating effect can lead to the deterioration of the hot spot, trigger molecular desorption, or initiate pyrolysis. These consequences may result in a decrease in the SERS signal and render it unstable [55].

The analyte molecule adsorbed on the surface of the PNP is excited by an enhanced field ${\bar{E}}_{L}$ of the laser, resulting in enhanced Raman-scattered light given by $E_{R}=\beta_{R}E_{0}{\bar{E}}_{p}$, where $\beta_{R}$ is the fraction of photons undergoing inelastic scattering, $E_{0}$ is the incident field, and ${\bar{E}}_{p}$ is the average field enhancement over the NP surface (i.e., the ratio of the enhanced field to the incident field). However, the SERS signal is proportional to the number of molecules in the hot spot [55].

(2) $$I_{SERS}=cN_{m}{\left|{\bar{E}}_{L}\right|}^{2}{\left|{\bar{E}}_{St.}\right|}^{2}\sigma_{R}.$$

where c is the molecule concentration, $N_{m}$ is the number of molecules, ${\bar{E}}_{St.}$ is the field enhancement at the Stokes frequency, and $\sigma_{R}$ is the Raman cross-section of the adsorbed molecules. Therefore, SERS changes with varying concentrations of analyte molecules and thermophoresis due to the temperature gradient in the solution. In short, the SERS signal can be controlled by temperature and molecule concentration.

### 4.5. Calibration Curve of Standard HER-II Solution

**Figure 6:**
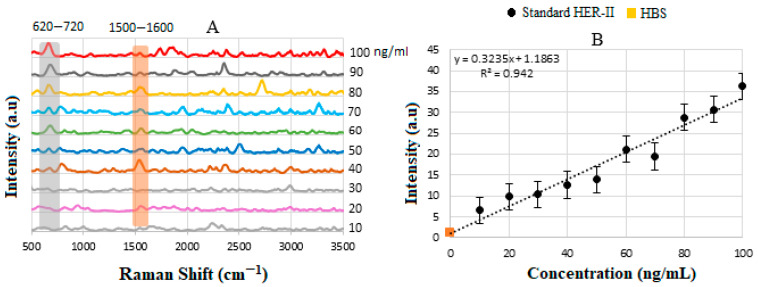
(**A**) Corresponding SERS spectra for the HER-II standard solution at various concentrations. The region 620−720 cm^−1^ indicates the linear increase in intensity with concentration, and the region 1500−1600 (cm^−1^) indicates a common feature in all of the cases but varies non-linearly. (**B**) Calibration curve for the standard HER-II biomarker based on Figure 4, with a sensitivity limit of about 10 ng/mL; the SERS intensity varies linearly with concentration.

Figure 6A illustrates the stacked SERS spectra for standard HER-II solutions at different concentrations between 10 and 100 ng/mL, where the overlapped peaks between 620 and 720 cm^−1^, shown by the grey stripe, indicate the presence of Tyr amino acids. Increasing the concentration of the sample increases the corresponding intensity linearly. Another region where the samples indicate a common response is between 1500 and 1600 cm^−1^, shown by a yellow stripe, which is related to secondary amines (stretching and bending) [36,56,57,58,59]. Both spectral regions can serve as reliable indicators for monitoring molecular changes; however, the first region demonstrates a linear response with analyte concentration, while the second exhibits a non-linear behavior. Consequently, the first spectral range was selected for constructing the calibration curve. Despite this, both regions offer valuable biomolecular insights. Each peak in the individual Raman spectrum shows the molecular composition at a specific location within the sample but may not represent other samples due to the absence of spectral superposition. By plotting the SERS signal intensities of characteristic peaks against the corresponding analyte concentrations, a calibration curve for the standard solution was generated, as shown in Figure 6B. The sensitivity of the SERS immunoassay was determined by the slope of this calibration curve. Healthy blood serum (HBS) was used as the reference baseline. The sensor substrate’s response uniformity was confirmed by the linearity observed throughout the experiment and the consistent monotonic increase in signal intensity with rising analyte concentration.

The slope of Figure 6B shows the dependence of SERS intensity on the analyte (HER-II) concentration c, which can be quantified as the rate of increase in the number of adsorbed analyte molecules ($N_{a}$) [60]:

(3) $$\frac{dN_{a}}{dt}=\left(1-\eta \right)k_{a}N_{w}-k_{d}N_{a}.$$

where $\frac{dN_{a}}{dt}$ shows the rate of adsorption, $k_{a}$ is the adsorption probability per unit of time experienced by the $N_{w}=c.\delta s$ molecules in the wetting layer, δ is the thickness, s is the apparent surface, $k_{d}$ is the desorption probability per unit of time experienced by the $N_{a}$ adsorbed molecules, $\left(1-\eta \right)$ is the fraction of the free adsorption sites, $\eta =N_{a}/N$ is the Langmuir fractional occupancy of the adsorption sites, N is the total number of adsorption sites of the sensor, and $\frac{dN_{a}}{dt}$ at t=0 represents a fresh sensor immersed in the serum of the given c, i.e., no analyte molecules are adsorbed on the surface of the GNU, and the surface coverage is assumed to be zero, so Equation (3) becomes

(4) $$\frac{dN_{a}}{dt}=k_{a}(c.s\delta ).$$

Equation (4) indicates the direct relationship between the analyte (i.e., HER-II biomarker) concentration and the rate of increase in adsorbed molecules, hence the higher SERS intensity. The SERS intensity at a known position is given by $I(c)=I_{i}[f\eta (c)]$, where $I_{i}$ is the initial maximum intensity, f is the fraction of the adsorption sites on the substrate determined by the regions of hot spots, and η=1 represents the maximum fractional occupancy as a function of c. The linear sensitivity for the standard HER-II solution from Figure 6B was obtained by the method of least squares as y=3031−1.5827 with $R^{2}=0.942$.

### 4.6. SERS of Conjugation and Interaction of BCS HER-II Samples

**Figure 7:**
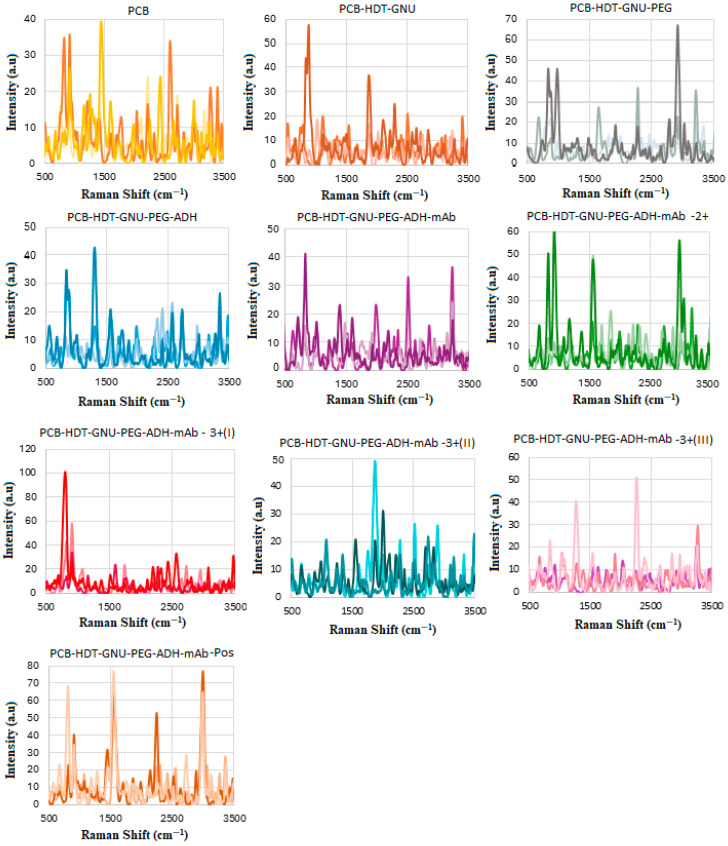
The trials of the SERS results of the functionalization, conjugation, and interaction steps for (GNU-mAb)-HER-II.

**Figure 8:**
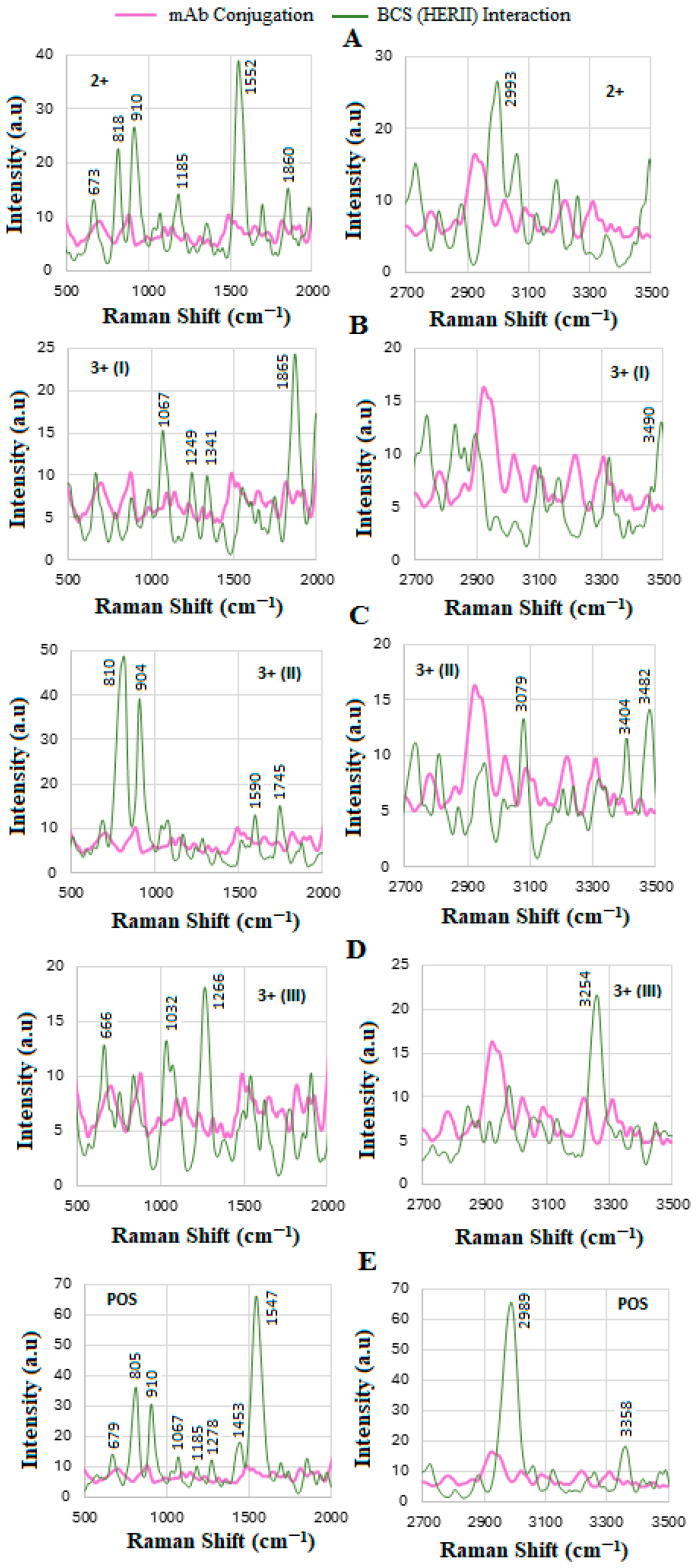
Comparison of SERS signals for conjugation and interaction of different types of HER-II BCS samples: (**A**) 2+, (**B**) 3+(I), (**C**) 3+(II), (**D**) 3+(III), and (**E**) POS.

The next phase involved conducting the SERS experiment using actual BCS samples of known types but unknown concentrations. Figure 7 presents the results of three SERS trials, corresponding to the functionalization, conjugation, and interaction steps. Figure 8 illustrates the SERS spectra before and after conjugation, highlighting both the fingerprint and high-wavenumber regions. Notably, the Raman signals become more defined and significantly enhanced following the interaction. The spectral profile and Raman line intensities vary according to the specific type of HER-II present. In our previous studies [39], where BCS’s interaction with GNU-conjugated mAbs was analyzed using NIR-FT spectroscopy, we observed a higher signal-to-noise ratio compared to the current SERS results. This discrepancy may be attributed to the relatively lower sensitivity of the Raman spectrometer used in this experiment.

The most dominant lines in Figure 8A for the 2+ sample include a weak peak at 673 cm^−1^, corresponding to Tyr, and relatively strong peaks at 818 and 910 cm^−1^, likely related to carbohydrate (-) ribose and glycine and alanine amino acids, respectively. It is also suggested that the range 900–1100 cm^−1^ is characterized by bands associated with several ν(C-C) and ν(C-N) stretching vibrations [58]. The peak at 1185 cm^−1^ shows exocyclic C-H and phenolic O-H bending motions in the plane of the ring, followed by a significant line at 1552 cm^−1^ indicating the presence of Trp, where normally the range 1550–1640 cm^−1^ is associated with N-H primary and secondary amines [57]. At the higher-wavenumber region, the line at 2993 cm^−1^ indicates highly overlapped bands that result from C-H stretching vibrations [55]. In the case of 3+ (I) in Figure 8B, the main lines at 1067, 1249, and 1341 cm^−1^, though relatively weak, likely correspond to several vibrations, such as CC in the phenyl ring [61], lysine [58], amide III [51,62], and Trp α-helix [57,62]. The strong line at 1865 cm^−1^ probably indicates the Raman thiol SH stretching bands in the silent region. The line at 3490 cm^−1^ corresponds to NH_2_ in aromatic amines, primary amines, and amides.

However, the most significant lines in Figure 8C, i.e., 3+ (II), belong to carbohydrates and glycine and alanine amino acids at 810 and 910 cm^−1^, respectively. The 910 cm^−1^ line may also be due to C-C stretching vibrations. The line at 1590 cm^−1^ corresponds to N-H primary and secondary amines, other conformation-sensitive amide II modes [57], or the presence of Tyr [62]. The line at 1745 cm^−1^ may be considered within the silent region. Figure 8D shows the results for 3+ (III), wherein the fingerprint region of the lines at 660, 1032, and 1266 cm^−1^ is related to the rocking CO_2_^–^ of valine, Phe, and amide III, respectively [58,63,64]. The dominant 3254 cm^−1^ line mainly represents OH stretching, as well as symmetric and antisymmetric vibrations of water in polysaccharides and protein [59]. In the case of positive HER-II in Figure 8E, the weak lines likely correspond to the following: 647 cm^−1^ to cysteine [58], 1067 cm^−1^ to His [58], 1185 cm^−1^ to C-H and phenolic O-H bending in the plane of the ring [57], 1278 cm^−1^ to α-helical domains in proteins contributing to the amide III band [57,63], and 1455 cm^−1^ to C-H deformation (polysaccharide and protein) [63]. The two relatively intense lines at 805 and 910 cm^−1^ represent Tyr and proline, respectively [58,62], and the strong 1547 cm^−1^ line is an excellent marker of amide II (β-sheet protein) and C-H bending or C=C stretching [36,43]. In the long-wavenumber region, the line at 2989 cm^−1^ is related to CH_3_, CH_2_, and =CH_2_ stretching of carbohydrates, lipids, and proteins [47,65] and that at 3358 cm^−1^, i.e., within 3330–3400 cm^−1^, corresponds to NH_2_ stretching in primary amines [55,61,66]. It seems that some lines are repeated features in the BCS samples, and that the content of NH_2_ in aromatic amines and primary amides as well as NH_3_^+^ in amino acids becomes important as the sample type advances. Some of the tentative corresponding assignments of the Raman shifts associated with mAbs and proteins are denoted in Table 4 [36,47,48,49,50,51,52,53,57,58,61,62,63,64,65,66].

### 4.7. Calibration Curve of BCS HER-II Samples

**Figure 9:**
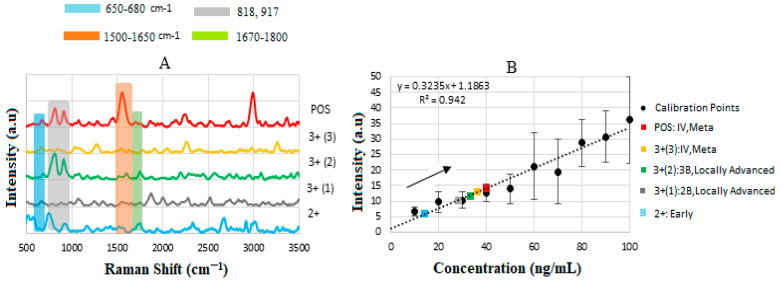
(**A**) Corresponding SERS spectra for various types of HER-II BCS samples. A linear increase in signal intensity is observed in the 650−680 cm−^1^ region, which is associated with specific molecular vibrations linked to HER-II expression. Other annotated spectral regions also provide relevant biochemical information but exhibit non-linear intensity variations. (**B**) The calibration curve was derived from the SERS intensities shown in (**A**) for HER-II BCS samples. This curve was used to estimate the unknown biomarker concentrations in clinical samples.

Figure 9A illustrates the corresponding SERS spectra obtained for the various types of BCS samples, where they exhibited similar characteristics and provided useful information in different regions. However, only the region between 650 and 680 cm^−1^ is used as the key feature to determine the corresponding concentration, due to the monotonic change in the intensity. The 650–680 cm^−1^ SERS peaks for HER-II are significant because they likely reflect aromatic amino acid vibrations and proteins commonly attributed to C–C twisting or ring breathing modes of phenylalanine. In the case of HER-II, such a signal could indicate the presence or conformational state of phenylalanine residues in the extracellular domain, serving as spectral markers for HER-II’s molecular structure and presence. In a diagnostic context, these peaks can help identify HER-II-positive cells with high specificity and sensitivity. When HER-II is targeted with SERS-active probes, as in our case, this spectral region may reflect binding interactions or molecular conformational changes, helping confirm specific attachment or expression levels. Interestingly, a consistent SERS peak in this range can act as a spectroscopic marker for the presence or overexpression of HER-II. Therefore, reliable detection in this region supports early, label-free cancer detection. The 1500–1650 cm^−1^ region in SERS corresponds to amide II due to N–H bending and C–N stretching. It also provides insight into the protein content, structure, and conformational changes, which is important for HER-II biomarker analysis in cancer diagnostics because it captures protein-specific vibrational signals, particularly from amide bonds. This range primarily arises from C=O stretching vibrations in the protein backbone. It is sensitive to the secondary protein structure (e.g., α-helix, β-sheet).

The sensitivity of SERS is discussed based on the slope of the calibration curve, which is the relationship between the SERS intensity and the concentration of the analyte, as shown in Figure 9B. The linear sensitivity was obtained by the method of least squares and was drawn as the best fit with a value of y = 0.324x + 1.186 and $R^{2}=0.942$, with a threshold value of 10 ng/mL. As can be seen, the data points are suitably positioned on the calibration curve; thus, by extrapolating each point to the *x*-axis, the corresponding concentration of the BCS sample can be determined. Notably, the measured concentration increases linearly with the HER-II classification level, indicating a correlation between disease progression and biomarker expression. In our case, the sensitivity of the calibration curve was between 10 and 100 ng/mL but the samples covered a range between 14 and 40 ng/mL for 2+ and positive, respectively; the results are tabulated in Table 5. Although the other spectral regions showed non-linearity, the biochemical information can be very useful; for example, the samples 2+, 3+ (II), and positive indicated high contents of carbohydrates and of glycine and alanine amino acids compared to 3+ (I) and 3+ (III). The regions 1550–1640 cm^−1^ and 2900–3000 cm^−1^, corresponding to N-H primary and secondary amines and amides (stretching and bending), along with O-H stretching (broad), are the most dominant peaks in the BCS positive sample. The discrepancies observed in Table 5 between a standard (or pure) biomarker and a real serum biomarker are due to the chemical environment, molecular interactions, and sample complexity, all of which significantly affect the SERS signal. For example, the standard biomarker is dissolved in clean buffer or water; the interactions are minimal, mostly free and unbound; and the effect on SERS is a strong and clean characteristic Raman signal. However, the real serum biomarkers present in complex biological fluids are bound to proteins (e.g., albumin, globulins), lipids, or vesicles, and the SERS results may be quenched, broadened, or even shifted.

### 4.8. SERS of Conjugation and Interaction of Standard CA15-3 Solution

**Figure 10:**
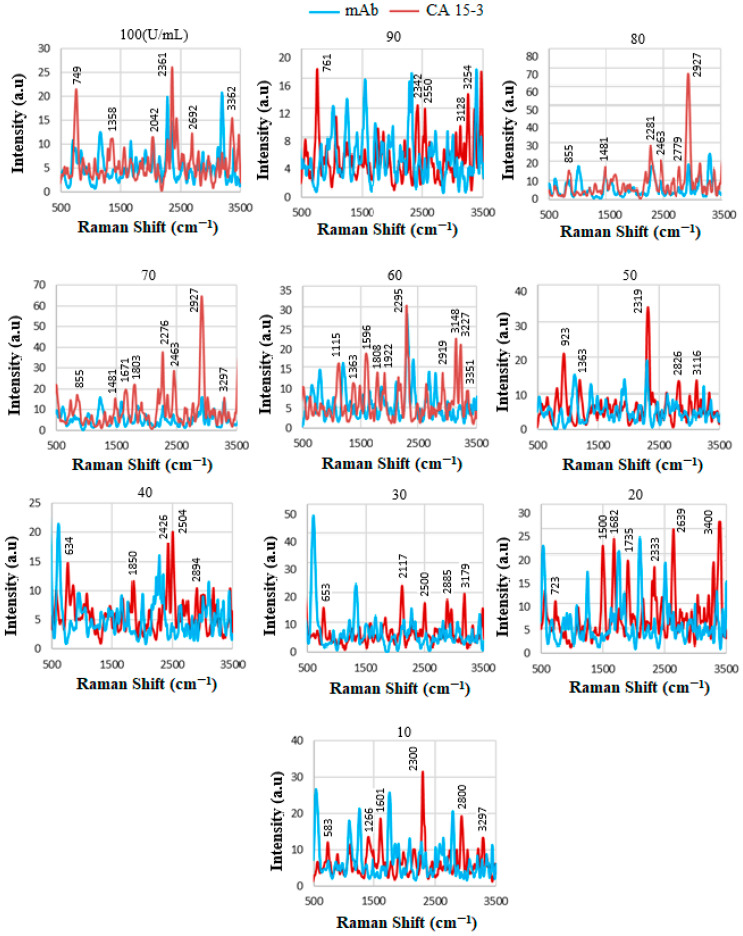
SERS signals of standard CA15-3 solutions for before (mAb-conjugated) and after interaction at various concentrations.

CA15-3 (Cancer Antigen 15-3) is a tumor-associated glycoprotein that is commonly used as a biomarker in the monitoring of breast cancer. Certain proteins, including those involved in inflammation and immunity, may increase in the serum of individuals with cancer. The overall composition of proteins in the serum can shift, with some proteins becoming more or less abundant. Figure 10 presents the spectral profiles of the mAb conjugate and its interactions with standard CA15-3 solutions at varying concentrations. The data demonstrate the successful detection of the CA15-3 biomarker, as evidenced by increased spectral intensity, which can be attributed to the presence of proteins and carbohydrates associated with the biomarker. Notably, the peak intensities exhibit a non-linear variation with increasing CA15-3 concentrations, suggesting differences in the amount and structural characteristics of protein contents present at each concentration level. This non-linear behavior may also be influenced by variations in binding efficiency and conformational changes of the mAb–antigen complex.

Several prominent vibrational bands can be observed across the spectra. These include the following: a band between approximately ≈640 and 650 cm^−1^, corresponding to Tyr vibrational modes; peaks in the range of ≈850–900 cm^−1^, which are attributed to the symmetric C-N-C stretching mode, amide II β-sheet protein structures, and primary N–H bending; a broad region from ≈2350 to 2750 cm^−1^, associated with NH_3_^+^ stretching vibrations; and strong absorptions above ≈3200 cm^−1^, corresponding to O-H stretching and N-H vibrational modes, indicative of hydrogen bonding and the presence of polar functional groups. These spectral features collectively support the presence and interaction of CA15-3 with the antibody conjugate, confirming its potential for use in sensitive and specific biomarker detection

**Figure 11:**
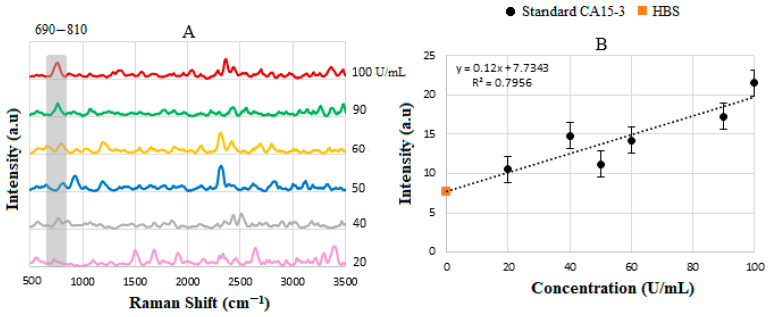
(**A**) Corresponding SERS spectra for CA15-3 standard solution at various concentrations. The region 690−810 cm−^1^ indicates the linear increase in intensity with concentration. (**B**) The calibration curve for the standard CA15-3 biomarker based on Figure 11A, with a sensitivity limit of about 10 ng/mL; the SERS intensity varies linearly with concentration.

The SERS spectra for the standard solutions are shown in Figure 11A. The binding of CA15-3 led to changes in the SERS signal, which were detected by a spectrometer. The intensity or shift in the signal correlates with the concentration of CA15-3, e.g., those between ≈2350 and 2500 cm^−1^ indicating NH_3_^+^, which is a protonated amino group in amino acids. Note that the biosensor operates based on the SV software, which utilizes the intensity value at the actual peak within an SERS range of 690–810 cm^−1^ (and not a fixed peak value) to plot the calibration line. Therefore, some of the peaks that were shifted were not considered for the plotting, as they did not show a peak in the range above. The intensities were then used to plot the corresponding calibration line in Figure 11B, with $R^{2}\approx 0.8$.

**Figure 12:**
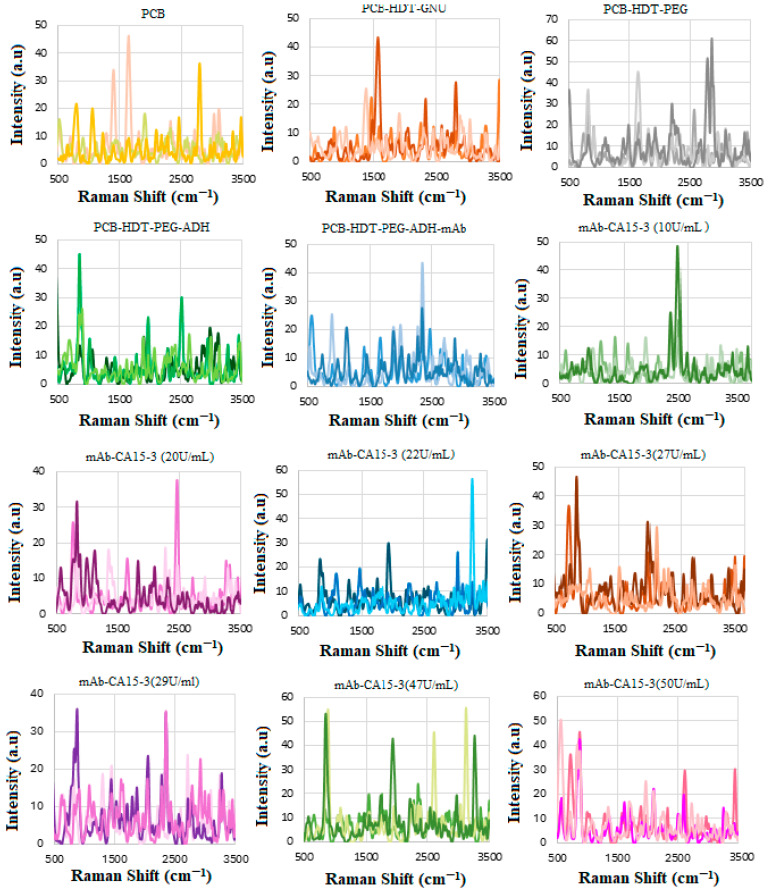
The trials of the SERS results of the functionalization, conjugation, and interaction steps for (GNU-mAb)-CA15-3.

**Figure 13:**
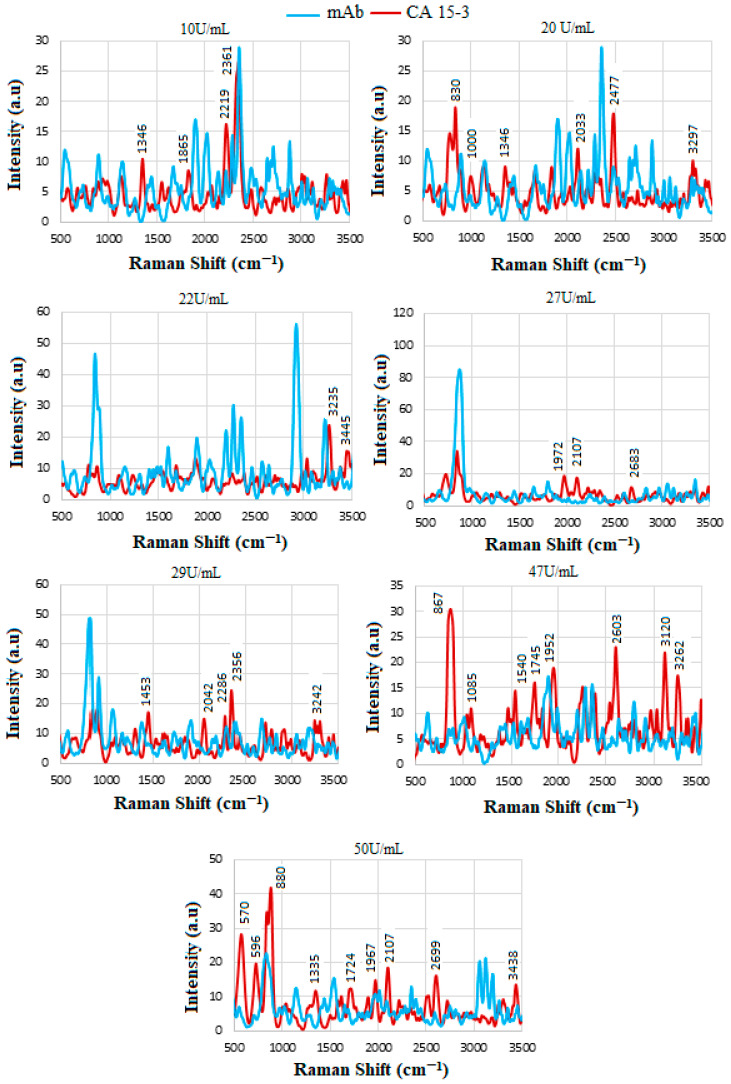
SERS signals of CA15-3 BCS samples for before (mAb-conjugated) and after interaction at various concentrations.

Figure 12 shows the stepwise procedure of the sensor preparation, including the three trials of functionalization, mAb conjugation, and interaction of the (GNU-mAb)-CA15-3 biomarker. This was then followed by plotting the average of each CA15-3 trial versus the mAb, as shown in Figure 13. The intensity of the signals varies randomly with the concentration, which could be due to several factors, including low concentrations in serum, especially in early-stage cancer. The variation in concentrations in individuals, depending on cancer stage, subtype, and physiological factors, can influence the SERS signal strength. Non-specific binding of some serum proteins (like albumin or globulins) to nanoparticles may block target sites or produce background signals. It is known that in serum, nanoparticles may agglomerate unpredictably due to ionic and protein interactions. This leads to inconsistent “hot spot” formation, where Raman enhancement occurs, resulting in variable intensity even for the same biomarker amount. In addition, in blood serum, steric hindrance and competitive binding reduce target capture, causing signal loss or inconsistency [36,39,67,68].

There are several alternatives that can be utilized by researchers to address the variability and background noise introduced by non-specific serum protein interactions and nanoparticle agglomeration in SERS. These include (a) PEGylation, where PEG molecules are used to create a hydrophilic, steric barrier around the nanoparticles, preventing non-specific protein adsorption; (b) nanoparticle engineering, including coating metal NPs with a thin dielectric shell (like silica), which can physically separate proteins from the metal surface while still allowing electromagnetic field penetration for SERS; (c) controlling the ionic strength of the medium by optimizing the salt concentration, which offers some help to manage NPs’ aggregation tendencies; and (d) integration of SERS into microfluidic platforms, allowing for the precise control of the sample flow and nanoparticle interaction conditions. The advantage of this system is mitigating sample complexity, hence enabling repeatable NP positioning and limiting serum-induced variability. Finally, the use of the training algorithms via advanced computational techniques such as machine learning and spectral correction can significantly distinguish target biomarker spectra from interfering protein signatures. A combination of these strategies offers a promising approach for advancing SERS toward more robust, reproducible, and clinically relevant biomarker detection, such as for HER-II in cancer diagnostics. The overlapped lines indicate the enhanced signals at specific sites, as summarized in Table 6.

**Figure 14:**
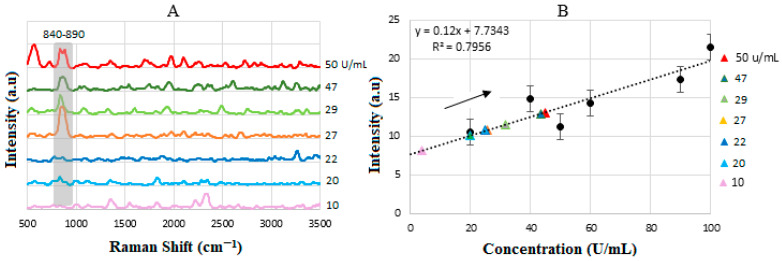
(**A**) Corresponding SERS spectra for various types of CA15-3 BCS samples. A linear increase in signal intensity is observed in the 640−890 cm−^1^ region, which is associated with specific molecular vibrations linked to CA15-3 expression. (**B**) The calibration curve was derived from the SERS intensities shown in (**A**) for CA-153 BCS samples. This curve was used to estimate the unknown biomarker concentrations in clinical samples.

Figure 14A presents the stacked SERS spectra of CA15-3, recorded at a range of concentrations. The spectral lines, particularly within the 840–890 nm region, show significant overlap, indicating consistent peak positions across different concentration levels. This spectral consistency reinforces the reproducibility of the measurement technique in this range. To further analyze the data, the corresponding SERS signal intensities were extracted and plotted against the known concentrations of CA15-3, as illustrated in Figure 14B. This plot serves to validate the quantitative accuracy of the spectral measurements by comparing the experimental results with the reference concentration values provided by the supplier. The correlation between intensity and concentration supports the reliability of the SERS-based detection method for CA15-3 quantification. The linear sensitivity was obtained by the method of least squares and was drawn as the best fit, with a value of y = 0.12x + 7.73 and $R^{2}=0.796$ with a threshold value of 20 U/mL. Table 7 summarizes the comparison between the actual and the SERS-measured values of CA 15-3.

## 5. Statistical Analysis

Principal Component Analysis (PCA) and Linear Discriminant Analysis (LDA) were employed to process and classify the spectral data, facilitating the visualization of distinctions between different sample groups. PCA transforms a set of correlated variables into a new set of linearly independent variables, known as principal components, which capture the most significant variance within the data. In our study, each Raman spectrum initially consisted of 3000 intensity values, corresponding to 3000 wavenumbers. To reduce dimensionality while preserving essential information, we applied Principal Component Analysis (PCA), transforming the original dataset into three principal components: P1, P2, and P3. These components capture the underlying correlations between spectral intensity and sample identity. The differences observed among the PCs provide valuable insights into the relationships between the spectra and their corresponding sample groups. Ideally, scans from the same sample group will form tight clusters in the PC plot, indicating high measurement precision and consistency within the group. Additionally, clear separation between clusters of different sample groups reflects strong spectral distinction, suggesting that the groups are readily distinguishable based on their Raman signatures. LDA, like PCA, is a dimensionality reduction technique; however, it is supervised and specifically designed to enhance class separability. LDA works by identifying a new axis or set of axes that maximizes the separation between predefined groups while minimizing variance within each group. By projecting the data onto these discriminant axes, LDA simplifies complex, high-dimensional datasets while retaining the most relevant information for distinguishing between classes, effectively filtering out less informative variability.

**Figure 15:**
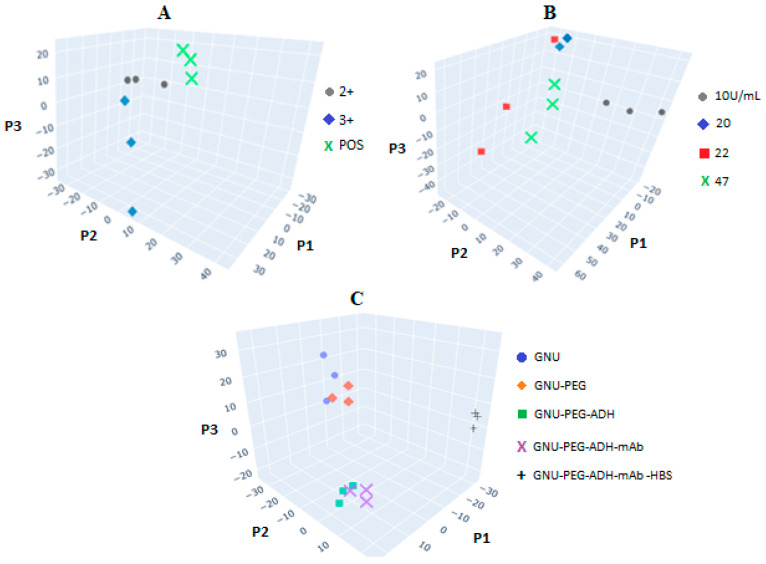
PCA plots for various stages of interaction: (**A**) GNU-mAb + HER-II, (**B**) GNU-mAb + CA15-3, and (**C**) GNU-mAb+HBS.

This process involves organizing the features into two or more groups to build a predictive model. In this context, the signal intensity values serve as the independent variables, while the sample identity functions as the dependent variable. Given the conceptual similarities between PCA and LDA, the principal component scores obtained from PCA can be directly used as input features for LDA. To evaluate the classification performance, a leave-one-out cross-validation (LOOCV) approach was employed, where one sample was omitted at a time and classified based on the remaining data. The results of the combined PCA-LDA analysis were visualized using principal component plots. Notably, LDA demonstrated perfect discrimination between the two groups, achieving 100% classification accuracy in all cases (i.e., a score of 1.0). Figure 15 presents the results of PCA applied to the SERS data obtained from the interaction of GNU-mAb with three different targets: HER-II (Figure 15A), CA 15-3 (Figure 15B), and healthy blood serum (HBS; Figure 15C). In each subplot, the PCA clearly distinguishes between the respective groups, indicating strong clustering and statistically significant separation. This distinct separation underscores the reproducibility and consistency of the individual SERS measurements across different sample types. The application of both LDA and PCA facilitates robust classification and highlights discernible differences in the spectral signatures. These differences are especially pronounced when comparing samples before and after interaction with the biomarkers or healthy serum. Such observable disparities confirm that the binding events and molecular interactions lead to characteristic spectral shifts, allowing for reliable identification and discrimination of the biomolecular targets. Collectively, these findings emphasize the utility of multivariate statistical techniques in enhancing the interpretability and diagnostic potential of SERS-based biosensing platforms.

## 6. Conclusions

Quantitative detection of HER-II and CA 15-3 biomarkers was successfully achieved using a dual-active SERS sensor substrate. Calibration curves were first established using the standard solution, with known concentrations of each biomarker. SERS spectra from unknown BCS samples were then recorded within specific wavenumber ranges and matched to the calibration data. The detection limits reached 10 ng/mL for HER-II and 20 U/mL for CA 15-3. The molecular band assignments varied based on biomarker type, concentration, and chemical structure. For HER-II, the SERS intensity increased in the order of 2+ > 3+(I) > 3+(II) > 3+(III) > Pos (IV), indicating a strong correlation with biomarker expression. Similarly, the CA 15-3 concentrations derived from spectral analysis were closely aligned with calibration curves. Principal Component Analysis–Linear Discriminant Analysis (PCA-LDA) revealed clear clustering of the sample groups, confirming the statistical robustness and reproducibility of the measurements. These results highlight the potential of SERS-based immunosensors for the simultaneous and accurate quantification of multiple cancer biomarkers. However, further optimization is needed to enhance sensitivity, improve substrate stability, increase sensitivity for early-stage detection, or integrate with other diagnostic modalities and adapt protocols for clinical use to guide future research. It is further recommended to explore the use of shorter linkers for the binding of GNUs and antibody conjugation. Utilizing shorter linkers may facilitate closer proximity and stronger interactions between the components, which can lead to a more efficient and pronounced enhancement of the SERS signals. Investigating these shorter linkers could improve the sensitivity and specificity of SERS-based detection methods, potentially advancing applications in biosensing and molecular diagnostics.

## Figures and Tables

**Table 1:** List of HER-II BCS samples used in the experiment.

Age.	HER-II (IHC Test)	CA 15-3 Concentration (U/mL)	Cancer Stage	Inventory Code
68	2+	14	Early	2399645
33	3+ (I)	22	IIB-Early	2372644
74	3+ (II)	N/A	IIIB-Locally Advanced	201608071
64	3+ (III)	N/A	IV-Meta	HUMANSRM-0004591
61	POS	N/A	IV-Meta	2572228

**Table 2:** List of CA15-3 BCS samples used in the experiment.

Age.	CA 15-3 Concentration (U/mL)	HER-II (IHC Test)	Cancer Stage	Inventory Code
75	10	Negative	IA-Early	2399632
72	20	Negative	IIIA-Locally Advanced	2722228
33	22	3+ (1)	IIB-Early	2372644
62	27	N/A	IV (Meta)	202105740
41	29	N/A	IIB-Early	HUMANSRM-)-104805
76	47	N/A	IIA-Early	2356746
51	50	N/A	IIIC-Locally Advanced	HUMANSRM-0118596

**Table 3:** AFM-measured values of GNU-immobilized substrate parameters.

Line	Rq (nm)	Ra (nm)	Rsk
Red	86.867	78.876	0.015
Green	39.405	33.045	0.914

**Table 4:** Some of the Raman shifts associated with some proteins.

Tentative Assignment	Wavenumber (cm^−1^)
S-S stretch disulfide	400–500
Ring modes of aromatic amino acids in proteins	505, 559
C-I stretch iodine derives	515, 850
C=O in amides	535–615
O-C-O in carboxylic acids	590–700
Phenylalanine	622
Tyrosine	642, 640–650
C-H out-of-plane bending vibration modes	675–850
Tryptophan, *δ*(-C-H)	722, 779
O-H out-of-plane bending	786
Tyrosine	830–840
Glycine, alanine v (C-N-C) assigned to the symmetric C-N-C stretching mode	850–900
C-H and phenolic O-H bending	950–1250
C-H, out-of-plane bending	965–980
Tyrosine	1000
Backbone vibrations	1006
Phenylalanine	1000–1010, 1103
C–O stretching	1034
C–C stretching in lipids	1076
Tyrosine ring breathing mode	1154
C–O stretching in lipids	1180
C–C stretching in lipids	1210
Amide III	1220
β-Sheet structures	1230–1245
Amide III bands	1245–1270
Phenolic C-O stretching	1260
C–C stretching in lipids	1267
Amide III bands	1260–1310
α-Helical domains related to the amide III band	1278
CH_2_ twisting and wagging in phospholipids	1302–1304
O-H in-plane vibration	1352
COO^−^ symmetric stretching: fatty acids, proteins	1392–1396
CH_2_ and CH_3_ deformation modes (lipids, proteins)	1440–1455
Protein amide II band	1507
COO^−^ stretching in carboxylates	1554
Amide II (β-sheet protein); N-H primary and secondary amines and amides (stretching and bending)	1547–1640
COO^-^ in carboxylic acid salts	1560–1610
NH_2_ in primary amines	1590–1650
C-O asymmetric stretching; -COO^−^ carboxylate	1610–1540
Tryptophan *ν*(C=C), tyrosine	1620
N-H in primary amides	1620–1650
C=O stretching in secondary amide (amide I)	1630–1680
C=O stretching in primary amide (amide I)	1650–1670
Amide I, α helix	1655–1665
β-Sheet structures	1665–1680
C=O stretching and N-H bending	1690–1700
C=O stretching in guanine	1700–1702
C=O stretching in lipids	1717, 1746
C-N amines, N-H primary and secondary amines and amides (stretch and bend), NH_3_ amine, and O-H stretching	1920–2300
N=C stretching	2115–2175
C≡C or C≡N stretching	2200–2260
N=C=O asymmetric Stretching in isocyanate	2271
P-H stretching	2280–2410
-NH_3_^+^ amine I	2350–2750
CH_2_ symmetric stretching in lipids	2854
CH_3_ symmetric stretching: proteins, lipids, triglycerides	2873–2880
CH_2_ asymmetric stretching in lipids and proteins	2890
CH_2_ asymmetric stretching	2921–2926
CH_2_ antisymmetric stretching in lipids	2934
O-H stretching in carbohydrates	2744
CH_2_ antisymmetric stretching in lipids	2882
C-H stretching: carbohydrates, lipids, proteins	2800–3300
CH_3_ asymmetric stretching: proteins, lipids, triglycerides	2957–2959
CH stretching in proteins	2996
O-H stretching	3200–3400
N-H vibration	3236
N-H stretching in primary amides, -NH_2_ in aromatic amines	3250–3500

**Table 5:** SERS-measured values of BCS HER-II type concentrations.

Sample	Measured Concentration (ng/mL)	Intensity (a.u)	Corresponding Stage
2+	14	6.0	Early
3+ (I)	28	10.24	IIB-Early
3+ (II)	33	11.95	IIIB- Loc. Adv.
3+ (III)	36	12.88	IV- Meta
POS	40	14.13	IV- Meta

**Table 6:** Summary of the SERS results based on Figure 13 for CA15-3 interaction.

U/mL	Wavenumber (cm^−1^)	Tentative Assignment
10	1346 2219	O-H in-plane vibration C≡C, C≡N stretching
20	2477 3297	NH_3_^+^ O-H stretching
22	3445	N-H stretching, NH_2_ aromatic amines
27	1972 2107 2683	C-N amines C-N amines NH_3_^+^
29	1453 2286 2356	CH_2_ and CH_3_ deformations O-H stretching -NH_3_^+^ I amine
47	867 1540 2603 3120 3262	Glycine, alanine Amide-II NH_3_^+^ amine C-H stretching N-H stretching, NH_2_ in aromatic amines
50	570 596 880 1967 3438	C=O in amides C=O in amides Glycine, alanine C-N amines, N-H primary N-H stretching in primary amides, NH_2_ in aromatic amines

**Table 7:** SERS-measured values of CA15-3 type concentrations.

Sample	Actual Concentration (U/mL)	Measured Concentration (U/mL)	Intensity (a.u)
1	10	4	8
2	20	19.70	10
3	22	24.5	10.75
4	27	25	10.9
5	29	31.7	11.4
6	47	43.5	12.85
7	50	45	13

## Data Availability

All data generated or analyzed during this study are included in this published article.

## References

[1] Loeb K.R., Loeb L.A. (2000). Significance of multiple mutations in cancer. Carcinogenesis.

[2] Siegel R.L., Jamel A., Wander R.C. (2018). An assessment of progress in cancer control. CA Cancer J. Clin..

[3] Fan L., Strasser-Weippl K., Li J., St Louis J., Finkelstein D., Chen W., Shao Z. (2014). Breast cancer in China. Lancet Oncol..

[4] Momenimovahed Z., Salehiniya H. (2019). Epidemiological characteristics of and risk factors for Breast cancer in the world. Breast Cancer.

[5] Zhang P., Xiao J., Ruan Y., Zhang Z., Zhang X. (2020). Monitoring value of serum HER2 as a predictive biomarker in patients with metastatic breast cancer. Cancer Manag. Res..

[6] Shaath H., Elango R., Alajez N.M. (2021). Molecular classification of breast cancer utilizing long non-coding RNA (lncRNA) tran-scriptomes identifies novel diagnostic lncRNA panel for triple-negative breast cancer. Cancers.

[7] Lipscomb J. (2008). Estimating the cost of cancer care in the United States: A work very much in progress. J. Natl. Cancer Inst..

[8] Kazarian A., Blyss O., Metodieva G., Gentry-Maharaj A., Ryan A., Kiseleva E.M., Prytomanova O.M., Jacobs I.J., Widschwendter M., Menon U. (2017). Testing breast cancer serum biomarkers for early detection and prognosis in pre-diagnosis samples. Br. J. Cancer.

[9] Manuali E., De Giuseppe A., Feliziani F., Forti K., Casciari C., Marchesi M.C., Pacifico E., Pawłowski K.M., Majchrzak K., Król M. (2012). CA 15–3 cell lines and tissue expression in canine mammary cancer and the correlation between serum levels and tumour histological grade. BMC Vet. Res..

[10] Zhu H., Dale P.S., Caldwell C.W., Fan X. (2009). Rapid and label-free detection of breast cancer biomarker CA15-3 in clinical human serum samples with optofluidic ring resonator sensors. Anal. Chem..

[11] Handy B. (2009). The clinical utility of tumor markers. Lab. Med..

[12] Fan X., White I.M., Shopova S.I., Zhu H., Suter J.D., Sun Y. (2008). Sensitive optical biosensors for unlabeled targets: A review. Anal. Chim. Acta.

[13] Stone N., Prieto M.C.H., Crow P., Uff J., Ritchie A.W. (2007). The use of Raman spectroscopy to provide an estimation of the gross biochemistry associated with urological pathologies. Anal. Bioanal. Chem..

[14] Silveira L., Leite K.R.W., Silveira F.L., Srougi M., Pacheco M.T.T., Zângaro R.A., Pasqualucci C.A. (2014). Discrimination of prostate carcinoma from benign prostate tissue fragments in-vitro by estimating the gross biochemical alterations through Raman spectroscopy. Lasers Med. Sci..

[15] Willets K., Van Dune R. (2007). Localized surface plasmon resonance spectroscopy and Sensing. Annu. Phys. Rev. Chem..

[16] Jain P.K., Lee K.S., El-Sayed I.H., El-Sayed M.A. (2006). Calculated absorption and scattering properties of gold nanoparticles of different size, shape, and composition: Applications in biological imaging and biomedicine. J. Phys. Chem. B.

[17] Shanbhag M.M., Manasa G., Mascarenhas R.J., Mondal K., Shetti N.P. (2023). Fundamentals of bio-electrochemical sensing. Chem. Eng. J. Adv..

[18] Menon S., Mathew M.R., Sam S., Keerthi K., Kumar K.G. (2020). Recent advances and challenges in electrochemical biosensors for emerging and re-emerging infectious diseases. J. Electroanal. Chem..

[19] Cao Y., Dai Y., Chen H., Tang Y., Chen X., Wang Y., Zhao J., Zhu X. (2019). Integration of fluorescence imaging and electro-chemical biosensing for both qualitative location and quantitative detection of cancer cells. Biosens. Bioelectron..

[20] Gil B., Keshavarz M., Wales D., Darzi A., Yeatman E. (2023). Orthogonal surface-enhanced Raman scattering/field-effect transistor detection of breast and colorectal cancer-derived exosomes using graphene as a tag-free diagnostic template. Adv. NanoBiomed Res..

[21] Gil Rosa B., Akingbade O.E., Guo X., Gonzalez-Macia L., Crone M.A., Cameron L.P., Freemont P., Choy K.-L., Güder F., Yeatman E. (2022). Multiplexed immunosensors for point-of-care diagnostic applications. Biosens. Bioelectron..

[22] Rodríguez-Oliveros R., Sánchez-Gill J. (2012). Gold nanostars as thermoplasmonic nanoparticles for optical heating. Opt. Express.

[23] Hrelescu C., Sau T.K., Rogach A.L., Jäckel F., Feldmann J. (2009). Single gold nanostars enhance Raman scattering. Appl. Phys. Lett..

[24] Shan F., Zhang X.-Y., Fu X.-C., Zhang L.-J., Su D., Wang S.-J., Wu J.-Y., Zhang T. (2017). Investigation of simultaneously existed Raman scattering enhancement and inhibiting fluorescence using surface modified gold nanostars as SERS probes. Sci. Rep..

[25] Jana D., Gorunmez Z., He J., Bruzas I.R., Beck T.L., Sagle L.B. (2016). Surface enhanced Raman spectroscopy of Au@Au core-shell structure containing a spiky shell. J. Phys. Chem. C.

[26] Khosroshahi M.E., Woll-Morison V., Patel Y. (2022). Near IR-plasmon enhanced guided fluorescence and thermal imaging of tissue subsurface target using ICG-labelled gold nanourchin and protein Contrast agent: Implication of stability. Lasers Med. Sci..

[27] Seungyoung P., Lee J., Ko H. (2017). Transparant and flexible SERS sensors based on gold nanostar array embedded in silicone rubber film. ACS Appl. Mater. Interfaces.

[28] Jalani G., Cerruti M. (2015). Nano graphene oxide-wrapped gold nanostars as ultrasensitive and stable SERS nanoprobes. Nanoscale.

[29] Lin J., Huang Z., Lin X., Wu Q., Quan K., Cheng Y., Zheng M., Xu J., Dai Y., Qiu H. (2020). Rapid and label-free urine test based on surface-enhanced Raman spectroscopy for the non-invasive detection of colorectal cancer at different stages. Biomed. Opt. Express.

[30] Kong K.V., Leong W.K., Lam Z., Gong T., Goh D., Lau W.K.O., Olivo M. (2014). A rapid and label-free SERS detection method for biomarkers in clinical biofluids. Small.

[31] Lin Y., Gao J., Tang S., Zhao X., Zheng M., Gong W., Xie S., Gao S., Yu Y., Lin J. (2021). Label-free diagnosis of breast cancer based on serum protein purification assisted surface-enhanced Raman spectroscopy. Spectrochim. Acta Part A Mol. Biomol. Spectrosc..

[32] Avci E., Yilmaz H., Sahiner N., Tuna B.G., Cicekdal M.B., Eser M., Basak K., Altıntoprak F., Zengin I., Dogan S. (2022). Label-free surface enhanced Raman spectroscopy for cancer detection. Cancers.

[33] Khosroshahi M.E., Chabok R., Chung N. (2023). Glass-based biosensor for targeted detection of HER-II biomarker in serum using oriented antibody-conjugated gold nanourchin. Plasmonics.

[34] Xia M., Zhang P., Qiao K., Bai Y., Xie Y.-H. (2016). Coupling SPP with LSPR for enhanced field confinement: A simulation study. J. Phys. Chem. C.

[35] Bhattarai J.K., Maruf M.H.U., Stine K.J. (2020). Plasmonic-active nanostructured thin films. Processes.

[36] Khosroshahi M.E., Patel Y., Chabok R. (2023). Non-invasive optical characterization and detection of CA 15-3 breast cancer bi-omarker in blood serum using monoclonal antibody-conjugated gold nanourchin and surface-enhanced Raman scattering. Lasers Med. Sci..

[37] Khosroshahi M.E., Patel Y., Umashanker V., Gaoiran C. (2024). Fabrication of and characterization of directional anti-body-conjugated gold nanourchin colloid and effect of laser polarization on SERS detection of breast cancer biomarker in serum. Colloids Surf. A Physicochem. Eng. Asp..

[38] Khosroshahi M.E., Patel Y. (2023). Reflective FT-NIR and SERS Study of HER-II breast cancer biomarker in blood serum using plasmonic-active nanostructured thin film immobilized with oriented monoclonal antibody. J. Biophotonics.

[39] Khosroshahi M.E., Patel Y., Umashanker V. (2024). Targeted FT-NIR and SERS Detection of Breast cancer HER-II biomarkers in blood serum using PCB-based plasmonic active nanostructured thin film label-free immunosensor immobilized with directional GNU-conjugated antibody. Sensors.

[40] Antosiewicz M.J., Shugar D. (2016). UV-vis spectroscopy of tyrosine side-groups in studies of protein structure, Part 2: Selected applications. Biophys. Rev..

[41] Fisher R.D., Ultsch M., Lingel A., Schaefer G., Shao L., Birtalan S., Sidhu S.S., Eigenbrot C. (2010). Structure of the complex between HER2 and an antibody paratope formed by side chains from tryptophan and serine. J. Mol. Biol..

[42] Terävä J., Tiainen L., Lamminmäki U., Kellokumpu-Lehtinen P.-L., Pettersson K., Gidwani K. (2019). Lectin nanoparticle assays for detecting breast cancer-associated glycovariants of cancer antigen 15-3 (CA15-3) in human plasma. PLoS ONE.

[43] Machado M., Guelli S.M.A.G.U., Ferreira Morgado A., Caldas P.G., Ptak F., Prioli R. (2016). Influence of cellulose fibers and fibrils on nanoscale friction in kraft paper. Cellulose.

[44] Esenturk E.N., Walker A.R.H. (2009). Surface-enhanced Raman scattering spectroscopy via gold nanostars. J. Raman Spectrosc..

[45] Weiss A., Haran G. (2001). Time-dependent single-molecule Raman scattering as a probe of surface dynamics. J. Phys. Chem. B.

[46] Miranda A.M., Castilho-Almeida E.W., Martins Ferreira E.H., Moreira G.F., Achete C.A., Armond R.A., Dos Santos H.F., Jorio A. (2014). Line shape analysis of the Raman spectra from pure and mixed biofuels esters compounds. Fuel.

[47] Chisanga M., Muhamadali H., Ellis D.I., Goodacre R. (2018). Surface-enhanced Raman scattering (SERS) in microbiology: Illumination and enhancement of the microbial world. Appl. Spectrosc..

[48] Ma M., Zhang J., Liu Y., Wang X., Han B. (2024). Advances in the clinical application of Raman spectroscopy in breast cancer. Appl. Spectrosc. Rev..

[49] Jenkins A.L., Larsen R.A., Williams T.B. (2005). Characterization of amino acids using Raman Spectroscopy. Spectrochim. Acta Part A Mol. Biomol. Spectrosc..

[50] Santos J.J., Leal J., Dias L.A.P., Toma S.H., Corio P., Genezini F.A., Katti K.V., Araki K., Lugão A.B. (2018). Bovine serum albumin conjugated gold-198 nanoparticles as model to evaluate damage caused by ionizing radiation to biomolecules. ACS Appl. Nano Mater..

[51] Miura T., Thomas G.J. (1995). Raman spectroscopy of proteins and their assemblies. Proteins: Structure, Function, and Engineering.

[52] Zhu G., Zhu X., Fan Q., Wan X. (2011). Raman spectra of amino acids and their aqueous solutions. Spectrochim. Acta Part A Mol. Biomol. Spectrosc..

[53] Li H., Thomas G.J. (1991). Studies of virus structure by Raman spectroscopy. Cysteine conformation and sulfhydryl interactions in proteins and viruses. 1. Correlation of the Raman sulfur-hydrogen band with hydrogen bonding and intramolecular geometry in model compounds. J. Am. Chem. Soc..

[54] McNay G., Eustace D., Smith W.E., Faulds K., Graham D. (2011). Surface-Enhanced Raman Scattering (SERS) and Surface-Enhanced Resonance Raman Scattering (SERRS): A review of applications. Appl. Spectrosc..

[55] Kang T., Hong S., Choi Y., Lee L.P. (2010). The effect of thermal gradients in SERS spectroscopy. Small.

[56] Chen H., Lin Z., Mo L., Wu T., Tan C. (2015). Near-infrared spectroscopy as a diagnostic tool for distinguishing between normal and malignant colorectal tissues. BioMed Res. Int..

[57] Lambert J. (1987). Chapter 10: Infrared and Raman Spectrometries: Vibrational Spectrometries. Introduction to Organic Spectroscopy.

[58] Smith B. (2017). An IR spectral interpretation potpourri: Carbohydrates and alkynes. Spectroscopy.

[59] Mo W., Ke Q., Zhou M., Xie G., Huang J., Gao F., Ni S., Yang X., Qi D., Wang A. (2023). Combined morphological and spectroscopic diagnostic of HER2 expression in breast cancer tissues based on label-free Surface-enhanced Raman scattering. Anal. Chem..

[60] Zanchi C., Giuliani L., Lucotti A., Pistaffa M., Trusso S., Neri F., Tommasini M., Ossi P.M. (2020). On the performance of laser-synthesized, SERS-based sensors for drug detection. Appl. Surf. Sci..

[61] Pérez A., Prada Y.A., Cabanzo R., González C.I., Mejía-Ospino E. (2018). Diagnosis of chagas disease from human blood serum using surface-enhanced Raman scattering (SERS) spectroscopy and chemometric methods. Sens. Bio-Sens. Res..

[62] Varga-Obieta E., Martinez-Espinosa J., Martinez-Zerga B., Aguilar-Lemarroy A., Gonzalez-Solfs J. (2016). Breast cancer detection based on serum sample surface enhanced Raman spectroscopy. Lasers Med. Sci..

[63] Fazio B., D’aNdrea C., Foti A., Messina E., Irrera A., Donato M.G., Villari V., Micali N., Maragò O.M., Gucciardi P.G. (2016). SERS detection of biomolecules at physiological pH via aggregation of gold nanorods mediated by optical forces and plasmonic heating OPEN. Sci. Rep..

[64] Guo C., Guo X., Chu W., Jiang N., Li H. (2019). Spectroscopic study of conformation changes of bovine serum albumin in aqueous environment. Chin. Chem. Lett..

[65] Hopkinson J., Newbery J., Twardowski J. (1988). Spectroscopic and Structural Studies of Biomaterials I: Proteins.

[66] Li Y., Wang Z., Mu X., Ma A., Guo S. (2017). Raman tags: Novel optical probes for intracellular sensing and imaging. Biotechnology.

[67] Bonifacio A., Cervo S., Sergo V. (2015). Label-free surface-enhanced Raman spectroscopy of biofluids: Fundamental aspects and diagnostic applications. Anal. Bioanal. Chem..

[68] Tan Y., Yan B., Xue L., Li Y., Luo X., Ji P. (2017). Surface-enhanced Raman spectroscopy of blood serum based of gold nanoparticles for the diagnosis of the squamous cell carcinoma. Lipids Health Dis..

